# Supplementary material for: Insights into the structure and function of the human organic anion transporter 1 in lipid bilayer membranes
Source: Sci Rep. 2022 Apr 29;12:7057. doi: 10.1038/s41598-022-10755-2 (PMC9054760; doi:10.1038/s41598-022-10755-2)
Supplement: Supplementary file 1 — Supplementary Information. [file 41598_2022_10755_MOESM1_ESM.pdf]

Electronic Supplementary Information for

Insights into the structure and function of the human Organic Anion Transporter 1 in lipid bilayer membranes

Angelika Janaszkiewicz,[1] Ágota Tóth,[1] Quentin Faucher,[1] Marving Martin,[1,2] Benjamin Chantemargue,[2] Chantal Barin-Le Guellec,[3] Pierre Marquet,[1,4] Florent Di Meo,[1,*]

1 U1248 Pharmacology & Transplantation, INSERM and Univ. Limoges, 87000 Limoges, France
2 InSiliBio, 1 avenue d'Ester, Ester Technopôle, 87000 Limoges, France
3 CHU de Tours, 2 Boulevard Tonnellé, 37044 Tours, France
4 Department of Pharmacology and Toxicology, CHU Limoges, F-87000 Limoges, France.

*** Corresponding Author**

Florent Di Meo – florent.di-meo@inserm.fr

INSERM U1248 IPPRITT, CBRS, Faculté de Médecine et Pharmacie,

Univ. Limoges,

2 rue du Prof. Descottes,

87000 Limoges

France

List of Supplementary Tables

Supplementary Table S1. *In vitro studies of site-directed mutagenesis performed on OAT1.* 4
Supplementary Table S2. *Identities and similarities scores between hOAT1 and template proteins used by automatic protein structural prediction tool I-TASSER to build initial model prior to MD simulations.* 5
Supplementary Table S3. *Total number of POPC (N_{POPC}), POPE (N_{POPE}), cholesterol (N_{Chol}), water (N_{water}) molecules and atoms (N_{atoms}) as well as initial PBC box sizes in MD simulations of substrate free (apo) hOAT1.* 6
Supplementary Table S4. *The list of MFS transporters used for PCA and tilt angle analysis.* 7
Supplementary Table S5. *Topological MFS core definition for the MFS dataset used in the present study as well as for hOAT1 model. For sake of readability, TMH definitions are split into (**A**) N- and (**B**) C-bundles.* 8
Supplementary Table S6. *The helicity of each transmembrane helix is displayed as the average of 3 replicas for each amino acid numbered from 1 as the first in a helix. Corresponding amino acids are listed in the last row.* 10
Supplementary Table S7. *Sum of H-bond fractions for each pair of helices. The average along the simulations and standard deviation are provided.* 11
Supplementary Table S8. *Inter-helical interactions captioned within a pair of the functional helices. The values represent H-bond fraction for which values above 1 highlight possible multiple hydrogen bonds. The tables represent interactions for systems: (A) POPC:POPE:Chol (2:1:1), (B) POPC:POPE (3:1), (C) POPC:Chol (3:1), (D) POPC. H-bond fraction above 0.1 were considered.* 12
Supplementary Table S9. *The hydrogen bonding network of charge-relay system split into interactions within N-bundle triad, C-bundle triad, between bundles (inter-bundle/inter-triad) and interaction of motifs with TMH11, TMH5 and TMH4. **(A)** The interactions averaged over 3 replicas of hOAT1 in OF state **(B)** interactions averaged over 3 replicas of hOAT1 (AF2) in IF state. Values above 1.0 represent possible multiple hydrogen bonds.* 16

List of Supplementary Figures

Supplementary Figure S1. *Sequence alignment of hOAT1, hGlut3 (PDBID: 5C65), rGlut5 (PDBID: 4YBQ), XylE (PDBID: 4GBY) using clustal omega algorithm. Picture was rendered using JalView 2.1 with conserved amino acid colored in a blue gradient from similar (light) to identical (dark).* 18
Supplementary Figure S2. *The plot of root means square deviation (Å) in POPC:POPE:Chol (2:1:1), POPC:Chol (3:1), POPC:POPE (3:1) and pure POPC membrane in time evolution (µs).* 19
Supplementary Figure S3. *The comparison of AF2 (IF) and present study hOAT1 (OF) models. The alignment is displayed by a bundle, similarly like RMSD plots below. The crucial distributions for conformation distances are plotted for present hOAT1 model with vertical line representing distance for AF2 model.* 20
Supplementary Figure S4. *(a) hOAT1 (both conformations) projected onto MFS conformational space formed by resolved MFS proteins. (b) Porcupine plot presenting PC2 (distinguishes OF and IF conformation) shows higher variability for C-bundle.* 21
Supplementary Figure S5. *Barplot representing distances of extracellular (Met358-Ser139) and intracellular (Val211-Gly446) distances stressed out and compared with previous hOAT1 model in IF conformation by Tsigelny et al.* [24]. 22
Supplementary Figure S6. *The evolution of tilt angles (clock-wise) divided by transmembrane helices of equilibrated trajectory in time for (A) POPC:POPE:Chol (2:1:1), (B) POPC:Chol (3:1), (C) POPC:POPE (3:1), (D) POPC.* 27
Supplementary Figure S7. *Comparison of A-, B-, and C-helices in AF2 (upper) and present study model (bottom) of hOAT1.* 28
Supplementary Figure S8. *The averaged along MD simulations contact maps of hOAT1 in (A) OF and (B) IF conformation divided by transmembrane helices* 29
Supplementary Figure S9. *Dynamic Cross Correlation Matrix (DCCM) of (A) hOAT1 OF and (B) hOAT1 IF (in POPC:POPE:Chol 2:1:1) divided by transmembrane helices.* 31
Supplementary Figure S10. *Cavity pore radii of hOAT1 averaged over 500 snapshots of equilibrated trajectory over z axis for (A) POPC:POPE:Chol (2:1:1); (B) POPC:Chol (3:1); (C) POPC:POPE (3:1); (D) POPC.* 33

Supplementary Figure S11. *Distribution of intra- (TM4-TM10; right column) and extracellular (TM7-TM10; left column) distances. The distances were considered as centers of mass (only backbones) of following residues: 36-40, 354-357 for TMH1-TMH7 and 207-210; 442-445 for TMH4-TMH10.* 34
Supplementary Figure S12. *Conformational space obtained by PCA. Projected PC1 and PC2 represent 39% and 19% of variability.* 35
Supplementary Figure S13. *Visualization of (a) PC1 and (b) PC2 (from Supplementary Figure S12) representing opening of the extracellular cavity of hOAT1. Both PCs display correlated movement of TMH2 (light grey) and TMH11 (dark grey).* 36
Supplementary Figure S14. *The contribution of each TMH into PC1, PC2 and PC3.* 37
Supplementary Figure S15. *Inter-bundle distance of E[X_6]R motifs in N- and C-bundle pictured along MD simulations of OF (left) and IF (right) hOAT1.* 38
Supplementary Figure S16. *The PCA projection of MFS conformational space including hOAT1 (OF) models in different lipid bilayers.* 39
Supplementary Figure S17. *The tilt angle profile (averaged over MD simulations) of hOAT1 (OF) in different lipid bilayers.* 41
Supplementary Figure S18. *PCA projected on all membrane conformational space of hOAT1 embedded in POPC:POPE:Chol (2:1:1) (blues), POPC:Chol (3:1) (greens), POPC:POPE (3:1) (yellows) and POPC (reds)* 43
Supplementary Figure S19. *Lipid density map of hOAT1 inserted into POPC:POPE:Chol (2:1:1). Upper and bottom leaflet of POPE (on the left) and cholesterol (on the right) are represented as density of P and O atom, respectively.* 45

1. Supplemental Tables

Supplementary Table S1. *In vitro* studies of site-directed mutagenesis performed on OAT1.

Amino acid change	Localization	Substate	Effects	Cell model (*in vitro*)	Reference
p.Leu30Ala	TMH1	PAH	Loss of expression	COS-7	[14]
p.Thr36Ala			Loss of substrate recognition		
p.Asn39Glu	TMH1	PAH	Loss of transport activity; no effect of cell surface expression	HeLa	[15]
p.Asn(39/56/92/97)Glu	TMH1/ECL	PAH	Loss of transport activity and cell surface expression		
p.Tyr230Ala/Phe	TMH5		Loss of membrane protein expression and diminution of transport activity		
p.Lys431Ala/Tyr	TMH10	CDF PAH	Diminution of membrane protein expression and transport activity	Xenopus Oocyte	[16]
p.Phe438Ala/Tyr	TMH10		Diminution of membrane protein expression and transport activity		
p.Tyr490Ala<br>p.Leu503Ala<br>p.Leu504Ala	TMH12	PAH	Loss of membrane protein expression and transport activity	COS-7 and LLC-PK1	[17]
p.Arg466Lys	TMH11	PAH	Decreased transport activity but no impact on expression	Xenopus Oocyte	[18]
p.Ser203Ala	TMH4	AFV CDF	Decreased transport activity	HEK293	[19]
p.Tyr353Ala<br>p.Tyr354Ala	TMH7	PAH	Loss of transport activity	COS7	[20]
p.Trp346Ala<br>p.Thr349Ala		/	Loss of transport activity and of the total expression		
p.Glu506Ala/Gln	ICH6	PAH	Decreased transport activity	COS-7	[21]
p.Leu512Ala					
p.Cys440Ala	TMH10				
p.Cys189Ala	TMH4	PAH	Decreased transport activity	CHO cell	[22]
p.Cys341Ala	TMH7				

p.Cys206Ser	TMH4				
p.Cys335Ala	TMH7				
p.Cys379Ala	TMH8	PAH	Decreased transport activity and protein expression	HeLa-mOAT1 cell	23
p.Cys427Ala	TMH10				
p.Cys434Ala					

Supplementary Table S2. Identities and similarities scores between hOAT1 and template proteins used by automatic protein structural prediction tool I-TASSER to build initial model prior to MD simulations.

**Pairs (PDB ID)**	**Local sequence alignment***		**Global sequence alignment***	
	Identity %	Similarity %	Identity %	Similarity %
***h*OAT1- *h*Glut3 (5C65)**	100/454 (22%)	159/454 (35%)	126/637 (19%)	195/637 (30.6%)
***h*OAT1- *r*Glut5 (4YBQ)**	108/477 (22.6%)	188/477 (39.4%)	118/645 (18.3%)	207/645 (32.1%)
***h*OAT1- XylE (4GBY)**	105/488 (21.5%)	187/488 (38.3%)	112/642 (17.4%)	198/642 (35.8%)

** Sequence alignment was done using Needleman-Wunsch algorithm (global) and Smith-Waterman algorithm (local) using the https://www.ebi.ac.uk webserver.*

Supplementary Table S3. Total number of POPC (N_{POPC}), POPE (N_{POPE}), cholesterol (N_{Chol}), water (N_{water}) molecules and atoms (N_{atoms}) as well as initial PBC box sizes in MD simulations of substrate free (*apo*) *h*OAT1.

**POPC:POPE:Chol ratio**			**1:0:0**	**3:1:0**	**3:0:1**	**2:1:1**
Lipids	N_{POPC}	Upper leaflet	187	147	159	110
		Lower leaflet	187	147	159	110
		Total	374	294	318	220
	N_{POPE}	Upper leaflet	-	49	-	55
		Lower leaflet	-	49	-	55
		Total	-	98	-	110
	N_{chol}	Upper leaflet	-	-	53	55
		Lower leaflet	-	-	53	55
		Total	-	-	106	110
Water	N_{water}		43493	43931	44340	44255
Box size	x		119.99	120.54	122.16	121.76
	y		120.04	120.66	125.17	125.25
	z		140.47	140.47	140.47	140.47
Natoms			189376	192222	192263	192922

Supplementary Table S4. The list of MFS transporters used for PCA and tilt angle analysis.

PDB ID	Transporter	Organism	Conformation	Method	Resolution	Reference
4YB9	GLUT5	*Bos taurus*	IF	X-Ray diffraction	3.20 Å	[1]
4ZYR	LacY	*E.coli*	IF	X-Ray diffraction	3.31 Å	[2]
4GBY	XylE	*E.coli*	OF	X-Ray diffraction	2.81 Å	[3]
4GBZ	XylE	*E.coli*	OF^{occ}	X-Ray diffraction	2.89 Å	[3]
4GC0	XylE	*E.coli*	OF	X-Ray diffraction	2.60 Å	[3]
4JA3	XylE	*E.coli*	IF^{occ}	X-Ray diffraction	3.80 Å	[4]
4JA4	XylE	*E.coli*	IF	X-Ray diffraction	4.20 Å	[4]
4QIQ	XylE	*E.coli*	IF	X-Ray diffraction	3.51 Å	[5]
4PYP	GLUT1	*Homo sapiens*	IF	X-Ray diffraction	3.17 Å	[6]
5EQG	GLUT1	*Homo sapiens*	IF	X-Ray diffraction	2.90 Å	[7]
5EQH	GLUT1	*Homo sapiens*	IF	X-Ray diffraction	2.99 Å	[7]
5EQI	GLUT1	*Homo sapiens*	IF	X-Ray diffraction	3.00 Å	[7]
4ZWB	GLUT3	*Homo sapiens*	OF^{occ}	X-Ray diffraction	2.40 Å	[8]
4ZWC	GLUT3	*Homo sapiens*	OF	X-Ray diffraction	2.60 Å	[8]
5C65	GLUT3	*Homo sapiens*	OF	X-Ray diffraction	2.65 Å	[8]
4YBQ	GLUT5	*Rattus norvegicus, Mus musculus*	OF	X-Ray diffraction	3.27 Å	[1]
4LDS	GlcP	*Staphylococcus epidermidis*	IF	X-Ray diffraction	3.20 Å	[9]
1PW4	GlpT	*E.coli*	IF	X-Ray diffraction	3.30 Å	[10]
3WDO	YajR	*E.coli*	OF	X-Ray diffraction	3.15 Å	[11]
2GFP	EmrD	*E.coli*	OF	X-Ray diffraction	3.50 Å	[12]
	OAT1	*Homo sapiens*	IF	Alpha-fold 2		[13]

Supplementary Table S5. Topological MFS core definition for the MFS dataset used in the present study as well as for *h*OAT1 model. For sake of readability, TMH definitions are split into (**A**) N- and (**B**) C-bundles.

(A)

**PDB ID/ System**	**TMH1**	**TMH2**	**TMH3**	**TMH4**	**TMH5**	**TMH6**
4YB9	26-43	72-91	101-118	126-146	163-182	193-211
4ZYR	12-29	48-67	77-94	108-128	141-160	167-185
4GBY	16-33	59-78	88-105	128-148	164-183	201-219
4GBZ	16-33	59-78	88-105	128-148	164-183	201-219
4GC0	16-33	59-78	88-105	128-148	164-183	201-219
4JA3	16-33	58-77	87-104	127-147	166-185	201-219
4JA4	16-33	56-75	87-104	127-147	166-185	201-219
4QIQ	16-33	56-75	86-103	127-147	166-185	200-218
4PYP	18-35	63-82	95-112	120-140	157-176	187-205
5EQG	18-35	63-82	95-112	120-140	157-176	187-205
5EQH	18-35	63-82	95-112	120-140	157-176	187-205
5EQI	18-35	63-82	95-112	120-140	157-176	187-205
4ZWB	14-31	65-84	93-110	119-139	155-174	185-203
4ZWC	14-31	65-84	93-110	119-139	155-174	185-203
5C65	14-31	65-84	93-110	119-139	155-174	185-203
4YBQ	19-36	72-91	100-117	128-148	161-180	192-210
4LDS	12-29	43-62	73-90	96-116	134-153	161-179
1PW4	33-50	64-83	94-111	121-141	160-179	189-207
3WDO	15-32	50-69	79-96	104-124	137-156	165-183
2GFP	14-31	44-63	74-91	98-118	134-153	158-176
OAT1	20-37	132-151	163-180	191-211	222-241	249-267

(B)

PDB ID/ System	TMH7	TMH8	TMH9	TMH10	TMH11	TMH12
4YB9	280-302	316-335	344-361	375-393	413-433	440-458
4ZYR	222-244	262-281	290-307	316-334	348-368	381-399
4GBY	279-301	316-335	343-360	374-392	407-427	443-461
4GBZ	279-301	316-335	343-360	374-392	407-427	443-461
4GC0	279-301	316-335	343-360	374-392	407-427	443-461
4JA3	279-301	316-335	344-361	372-390	408-428	444-462
4JA4	279-301	317-336	344-361	372-390	409-429	444-462
4QIQ	281-303	315-334	344-361	372-390	409-429	444-462
4PYP	275-297	308-327	337-354	367-385	405-425	432-450
5EQG	275-297	308-327	337-354	367-385	405-425	432-450
5EQH	275-297	308-327	337-354	367-385	405-425	432-450
5EQI	275-297	308-327	337-354	367-385	405-425	432-450
4ZWB	272-294	306-325	335-352	366-384	402-422	430-448
4ZWC	272-294	306-325	335-352	366-384	402-422	430-448
5C65	272-294	306-325	335-352	367-385	402-422	430-448
4YBQ	277-299	317-336	345-362	370-388	411-431	439-457
4LDS	244-266	277-296	307-324	336-354	375-395	402-420
1PW4	254-276	292-311	323-340	350-368	386-406	417-435
3WDO	214-236	250-269	280-297	306-324	340-360	368-386
2GFP	207-229	239-258	267-284	291-309	326-346	358-376
OAT1	335-357	370-389	397-414	425-443	457-477	486-504

Supplementary Table S6. The helicity of each transmembrane helix is displayed as the average of 3 replicas for each amino acid numbered from 1 as the first in a helix. Corresponding amino acids are listed in the last row.

Number of amino acid	TM1	TM2	TM3	TM4	TM5	TM6	TM7	TM8	TM9	TM10	TM11	TM12
1	0.0	0.0	0.0	0.0	0.0	0.0	0.0	0.0	0.0	0.0	0.0	0.0
2	0.961	0.331	0.949	0.992	0.153	0.352	0.199	0.963	0.996	0.541	0.0	0.702
3	0.963	0.594	0.988	1.0	0.338	0.36	0.295	0.992	0.999	0.569	0.0	0.749
4	0.963	0.862	0.999	1.0	0.461	0.972	0.629	0.996	1.0	0.917	0.996	0.749
5	0.358	0.994	1.0	1.0	0.517	0.973	0.634	0.998	1.0	0.965	0.996	0.947
6	0.0	0.979	1.0	1.0	0.996	0.999	0.989	0.999	1.0	0.953	1.0	0.994
7	0.0	0.98	1.0	1.0	0.999	0.998	0.99	0.926	1.0	0.593	1.0	0.997
8	0.247	0.96	1.0	1.0	0.999	0.841	1.0	0.915	1.0	0.209	1.0	0.998
9	0.533	0.954	1.0	1.0	1.0	0.937	1.0	0.836	1.0	0.028	1.0	0.996
10	0.997	0.999	1.0	0.999	0.999	0.996	1.0	0.017	1.0	0.571	1.0	0.825
11	1.0	1.0	1.0	1.0	0.994	0.996	1.0	0.947	1.0	0.85	1.0	0.844
12	1.0	0.923	1.0	1.0	0.989	1.0	1.0	0.979	1.0	0.891	1.0	0.948
13	1.0	0.866	1.0	1.0	0.986	0.999	1.0	0.994	0.999	0.937	1.0	0.946
14	1.0	0.995	0.999	1.0	0.956	0.992	1.0	0.999	0.999	0.964	0.999	0.874
15	1.0	0.996	1.0	1.0	0.928	0.999	1.0	0.997	0.997	0.939	0.995	0.393
16	0.999	0.999	1.0	1.0	0.929	0.997	1.0	0.992	0.938	0.928	0.98	0.0
17	1.0	0.998	1.0	1.0	0.756	0.997	0.999	0.958	0.867	0.855	0.96	
18	0.997	1.0	1.0	1.0	0.734	0.996	0.957	0.954	0.0	0.607	0.801	
19	0.974	1.0	0.959	1.0	0.526	0.943	0.984	0.65		0.623	0.334	
20	0.952	1.0	0.824	1.0	0.0	0.853	0.971	0.634		0.995	0.248	
21	0.0	1.0	0.0	1.0		0.684	0.965	0.373		0.986	0.947	
22		0.999		0.999		0.0	0.864	0.0		0.951	0.999	
23		0.998		0.998			0.373			0.778	1.0	
24		0.993		0.913			0.954			0.0	1.0	
25		0.608		0.487			0.997				1.0	
26		0.991		0.418			0.989				1.0	
27				0.0			0.972				0.996	
28											0.996	
29											0.0	
Helices definition	19-39	127-156	161-181	186-212	220-239	247-268	333-361	368-389	395-412	422-445	451-481	488-503

Supplementary Table S7. Sum of H-bond fractions for each pair of helices. The average along the simulations and standard deviation are provided.

	TMH1	TMH2	TMH3	TMH4	TMH5	TMH6	TMH7	TMH8	TMH9	TMH10	TMH11	TMH12
TMH1		0.993 ±0.334	0.0±0.0	1.493 ±0.101	0.227 ±0.067	1.107 ±0.242	0.41 ±0.537	0.0±0.0	0.0±0.0	0.0±0.0	0.604 ±0.32	0.0±0.0
TMH2	0.993 ±0.334		0.289 ±0.238	0.266 ±0.209	0.0±0.0	0.002 ±0.004	0.0±0.0	0.0±0.0	0.0±0.0	0.0±0.0	0.476 ±0.458	0.0±0.0
TMH3	0.0±0.0	0.289 ±0.238		2.678 ±0.241	0.0±0.0	1.679 ±0.469	0.0±0.0	0.0±0.0	0.0±0.0	0.0±0.0	0.013 ±0.009	0.0±0.0
TMH4	1.493 ±0.101	0.266 ±0.209	2.678 ±0.241		0.438 ±0.121	0.593 ±0.063	0.0±0.0	0.0±0.0	0.0±0.0	0.056 ±0.045	0.013 ±0.019	0.013 ±0.019
TMH5	0.227 ±0.067	0.0±0.0	0.0±0.0	0.438 ±0.121		0.266 ±0.274	0.612 ±0.429	2.582 ±0.574	0.0±0.0	1.154 ±0.194	0.004 ±0.003	0.0±0.0
TMH6	1.107 ±0.242	0.002 ±0.004	1.679 ±0.469	0.593 ±0.063	0.266 ±0.274		0.0±0.0	0.0±0.0	0.0±0.0	0.0±0.0	0.0±0.0	0.0±0.0
TMH7	0.41 ±0.537	0.0±0.0	0.0±0.0	0.0±0.0	0.612 ±0.429	0.0±0.0		0.0±0.0	0.609 ±0.467	0.512 ±0.369	0.316 ±0.213	0.014 ±0.018
TMH8	0.0±0.0	0.0±0.0	0.0±0.0	0.0±0.0	2.582 ±0.574	0.0±0.0	0.0±0.0		0.007 ±0.01	0.424 ±0.255	0.0±0.0	0.0±0.0
TMH9	0.0±0.0	0.0±0.0	0.0±0.0	0.0±0.0	0.0±0.0	0.0±0.0	0.609 ±0.467	0.007 ±0.01		2.464 ±0.668	0.106 ±0.045	1.301 ±0.577
TMH10	0.0±0.0	0.0±0.0	0.0±0.0	0.056 ±0.045	1.154 ±0.194	0.0±0.0	0.512 ±0.369	0.424 ±0.255	2.464 ±0.668		2.222 ±0.614	0.944 ±0.015
TMH11	0.604 ±0.32	0.476 ±0.458	0.013 ±0.009	0.013 ±0.019	0.004 ±0.003	0.0±0.0	0.316 ±0.213	0.0±0.0	0.106 ±0.045	2.222 ±0.614		0.043 ±0.052
TMH12	0.0±0.0	0.0±0.0	0.0±0.0	0.013 ±0.019	0.0±0.0	0.0±0.0	0.014 ±0.018	0.0±0.0	1.301 ±0.577	0.944 ±0.015	0.043 ±0.052	

Supplementary Table S8. Inter-helical interactions captioned within a pair of the functional helices. The values represent H-bond fraction for which values above 1 highlight possible multiple hydrogen bonds. The tables represent interactions for systems: (A) POPC:POPE:Chol (2:1:1), (B) POPC:POPE (3:1), (C) POPC:Chol (3:1), (D) POPC. H-bond fraction above 0.1 were considered.

(A) POPC:POPE:Chol (2:1:1)

		rep1	rep2	rep3
A-helices	TMH1 - TMH7	-	Ala42-Tyr354 (0.856)<br>Thr36-Tyr353 (0.277)	-
	TMH4 - TMH10	-	Thr445-Gly457 (0.115)	-
B-helices	TMH2 - TMH11	Gln138-Ser472 (0.367)<br>Asp157-Gln455 (0.674)	-	Arg131-Glu480 (0.245)
	TMH5 - TMH8	Thr224-Asn390 (1.024)<br>Ala238-Gln371 (0.846)<br>Ala241-Gln371 (0.669)	Thr224-Asn390 (1.195)<br>Ser231-Asp378 (0.817)<br>Tyr228-Leu379 (0.271)<br>Ala238-Gln371 (0.737)<br>Ser231-Lys382 (0.122)	Tyr228-Lys382 (0.119)<br>Ser231-Asp378 (0.102)<br>Ala238-Gln371 (0.786)<br>Thr224-Asn390 (0.229)<br>Ala241-Gln371 (0.124)<br>Ser231-Lys382 (0.332)<br>Ala220-Asn390 (0.104)
C-helices	TMH3 - TMH6	Arg162-Phe268 (0.519)<br>Arg162-Glu270 (0.495)<br>Arg162-Trp266 (0.248)	Arg161-Ile269 (0.4)<br>Arg162-Trp266 (0.483)<br>Arg162-Phe268 (0.432)<br>Arg162-Glu270 (0.577)<br>Arg161-Glu270 (0.334)	Arg162-Trp266 (0.669)<br>Arg162-Glu270 (0.534)
	TMH9 - TMH12	Arg395-Leu504 (0.397)<br>Arg395-Val502 (0.151	Arg395-Leu504 (0.56)<br>Gln398-Thr501 (0.302)<br>Arg395-Thr501 (0.385)	Arg395-Leu503 (1.12)<br>Arg394-Leu504 (0.845)

(B) POPC:POPE (3:1)

		rep1	rep2	rep3
A-helices	TMH1 - TMH7	-	Asn39-Tyr353 (0.227)<br>Asn39-Tyr354 (0.576)	-
	TMH4 - TMH10	-	-	-
B-helices	TMH2 - TMH11	Asp157-Gln455 (0.185)	Gln135-Pro473 (0.213)<br>Gln138-Ser472 (0.157)<br>Phe152-Gln455 (0.516)	Gln138-Ser476 (0.626)<br>Asp157-Gln455 (0.214)
	TMH5 - TMH8	Ser231-Asp378 (0.895)<br>Leu237-Gln371 (0.795)<br>Gly227-Lys382 (0.249)	Gln234-Phe374 (0.222)<br>Ser231-Asp378 (0.197)<br>Ala238-Gln371 (0.287)<br>Gly227-Lys382 (0.203)<br>Ala241-Gln371 (0.104)	Ser231-Asp378 (0.966)<br>Tyr242-Gln371 (0.333)<br>Gln234-Asp378 (0.241)
C-helices	TMH3 - TMH6	Arg162-Trp266 (0.704)<br>Arg162-Phe268 (0.297)<br>Arg162-Glu270 (0.398)<br>Arg161-Glu270 (0.252)	Arg162-Trp266 (0.654)<br>Arg162-Glu270 (0.766)<br>Arg162-Phe268 (0.432)<br>Tyr169-Phe258 (0.218)<br>Tyr169-Phe259 (0.12)	Arg162-Trp266 (0.728)<br>Arg162-Glu270 (0.674)<br>Arg162-Phe268 (0.306)<br>Lys163-Glu270 (0.14)
	TMH9 - TMH12	Arg395-Leu504 (0.774)<br>Gln398-Thr501 (0.344)<br>Met399-Ser498 (0.221)	Arg395-Leu504 (0.827)<br>Gln398-Thr501 (0.218)<br>Met399-Ser498 (0.134)	Arg395-Leu504 (0.603)<br>Arg395-Thr501 (0.114)

(C) POPC:Chol (3:1)

		rep1	rep2	rep3
A-helices	TMH1 - TMH7	-	Asn39-Tyr353 (0.261)<br>Asn39-Tyr354 (0.17)<br>Asn35-Tyr354 (0.439)	Asn39-Tyr354 (0.261)
	TMH4 - TMH10	-	Hie217-Glu447 (0.419)	-
B-helices	TMH2 - TMH11	Gly153-Thr456 (0.56)<br>Asp157-Gln455 (0.116)	Arg134-Glu480 (1.196)<br>Gln138-Ser476 (0.304)<br>Gln135-Glu480 (0.323)<br>Gln135-Ser476 (0.132)<br>Arg131-Glu480 (0.127)	Gln135-Pro473 (0.185)<br>Asp157-Gln455 (0.414)
	TMH5 - TMH8	Thr224-Asn390 (1.068)<br>Ser231-Asp378 (0.941)<br>Ala238-Gln371 (0.425)	Ser231-Asp378 (0.966)<br>Thr224-Asn390 (0.943)<br>Tyr228-Leu379 (0.262)<br>Ala238-Gln371 (0.717)	Ser231-Asp378 (0.967)<br>Tyr228-Lys382 (0.452)<br>Leu237-Gln371 (0.71)
C-helices	TMH3 - TMH6	Tyr169-Phe259 (0.212)<br>Arg162-Glu270 (0.693)<br>Arg162-Trp266 (0.205)	Tyr169-Phe262 (0.591)<br>Arg162-Trp266 (0.435)<br>Arg162-Glu270 (0.825)<br>Tyr169-Ser265 (0.221)<br>Arg162-Phe268 (0.239)	Arg162-Phe268 (0.771)<br>Arg162-Glu270 (0.764)<br>Lys163-Glu270 (0.37)
	TMH9 - TMH12	Arg395-Leu504 (0.728)<br>Arg395-Val502 (0.158)	Arg395-Leu504 (1.347)	Arg395-Leu504 (0.92)

(D) POPC

		rep1	rep2	rep3
A-helices	TMH1 - TMH7	-	-	Asn35-Tyr354 (0.251)
	TMH4 - TMH10	-	-	-
B-helices	TMH2 - TMH11	Gln138-Pro473 (0.664)<br>Arg134-Glu480 (1.366)<br>Gln135-Glu480 (0.674)<br>Gln135-Ser476 (0.3)	Asp157-Gln455 (0.627)	Val145-Arg466 (0.525)<br>Gly153-Thr456 (0.158)<br>Ala156-Gln455 (0.157)
	TMH5 - TMH8	Ser231-Asp378 (0.966)<br>Ala238-Gln371 (0.619)	Tyr228-Leu379 (0.678)<br>Ala238-Gln371 (0.208)<br>Gly239-Gln371 (0.148)<br>Thr224-Lys382 (0.231)	Thr224-Asn390 (0.954)<br>Tyr228-Leu379 (0.383)<br>Ser231-Asp378 (0.545)<br>Ala238-Gln371 (0.397)<br>Ser231-Lys382 (0.128)
C-helices	TMH3 - TMH6	Arg162-Trp266 (0.465)<br>Arg162-Phe268 (0.376)<br>Arg162-Glu270 (0.349)<br>Arg161-Glu270 (0.47)<br>Arg162-Phe267 (0.126)	Arg162-Glu270 (0.561)<br>Arg162-Trp266 (0.465)<br>Arg162-Phe268 (0.108)	Arg162-Phe268 (0.613)<br>Arg162-Glu270 (0.757)<br>Arg162-Trp266 (0.292)
	TMH9 - TMH12	Arg395-Leu504 (0.785)	Arg395-Leu504 (0.77)	Arg395-Leu504 (1.076)<br>Gln417-Met485 (0.144)

Supplementary Table S9. The hydrogen bonding network of charge-relay system split into interactions within N-bundle triad, C-bundle triad, between bundles (inter-bundle/inter-triad) and interaction of motifs with TMH11, TMH5 and TMH4. **(A)** The interactions averaged over 3 replicas of *h*OAT1 in OF state **(B)** interactions averaged over 3 replicas of *h*OAT1 (AF2) in IF state. Values above 1.0 represent possible multiple hydrogen bonds.

(A)

	**N-bundle**		
	A-motif	E[X6]R	[P/X]ESRW[L/X]
A-motif	-	**Arg161-Glu212** (1.518)	Arg162-Glu270 (0.569)<br>Arg161-Ile269 (0.126)<br>**Arg161-Glu270** (0.109)
E[X6]R	-	-	Glu212-Arg273 (1.16)<br>**Glu212-Ser271** (0.620)
[P/X]ESRW[L/X]	-	-	-
	**C-bundle**		
	A-motif	E[X6]R	[P/X]ESRW[L/X]
A-motif	-	**Arg394-Glu447** (1.129)	**Arg394-Glu506** (0.674)<br>**Arg395-Glu506** (0.322)
E[X6]R	-	-	Glu447-Thr507 (0.630)
[P/X]ESRW[L/X]	-	-	-
	**Inter-bundle**		
	A-motif	E[X6]R	[P/X]ESRW[L/X]
A-motif	-	-	-
E[X6]R	-	Arg219-Glu447 (1.334)	-
[P/X]ESRW[L/X]	-	-	-
	**Motifs to TMH11/5/4**		
	TMH11	TMH5	TMH4
A-motif	Asp157-Gln455 (0.224)	Thr224-Asn390 (0.776)	Arg161-Asn205 (0.207)
E[X6]R	Glu212-Gln455 (0.464)	Ala220-Asn390 (0.134)	-

(B)

		**N-bundle**	
	A-motif	E[X6]R	[P/X]ESRW[L/X]
A-motif	-	Arg161-Glu212 (1.984)	Arg162-Glu270 (0.738)<br>Arg161-Glu270 (0.351)<br>Asp157-Trp274 (0.707)
E[X6]R	-	-	Trp213-Glu270 (0.186)<br>Trp213-Ile269 (0.126)<br>Glu212-Ser271 (0.997)<br>Glu212-Arg273 (0.194)
[P/X]ESRW[L/X]	-	-	-
		**C-bundle**	
	A-motif	E[X6]R	[P/X]ESRW[L/X]
A-motif	-	Arg394-Glu447 (1.948)	Arg394-Glu506 (0.821)<br>Arg395-Glu506 (0.532)
E[X6]R	-	-	Glu447-Thr507 (0.982)
[P/X]ESRW[L/X]	-	-	-
		**Inter-bundle**	
	A-motif	E[X6]R	[P/X]ESRW[L/X]
A-motif	-	-	-
E[X6]R	-	-	-
[P/X]ESRW[L/X]	-	-	-
		**Motifs to TMH11/5/4**	
	TMH11	TMH5	TMH4
A-motif	-	-	-
E[X6]R	-	-	-

2. Supplemental Figures

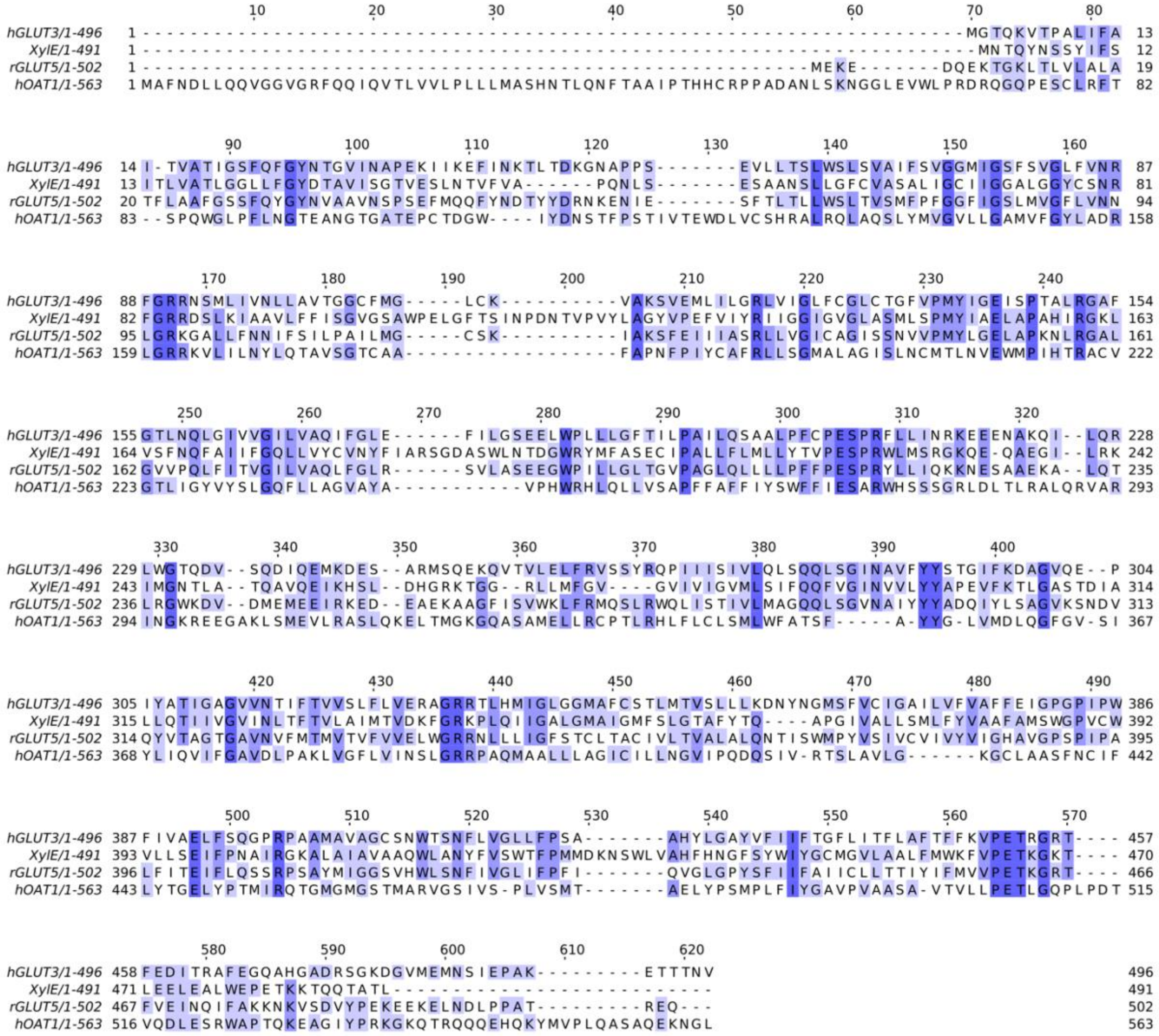

Supplementary Figure S1. Sequence alignment of *h*OAT1, *h*Glut3 (PDBID: 5C65), *r*Glut5 (PDBID: 4YBQ), XylE (PDBID: 4GBY) using clustal omega algorithm. Picture was rendered using JalView 2.1 with conserved amino acid colored in a blue gradient from similar (light) to identical (dark).

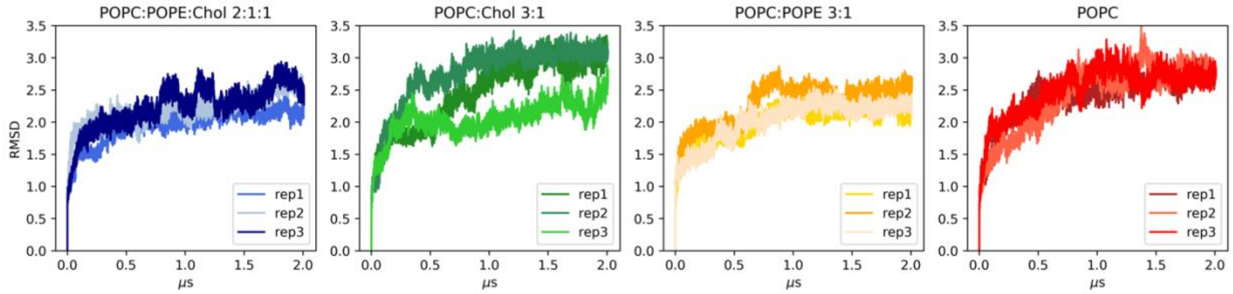

Supplementary Figure S2. The plot of root means square deviation (Å) in POPC:POPE:Chol (2:1:1), POPC:Chol (3:1), POPC:POPE (3:1) and pure POPC membrane in time evolution (μs).

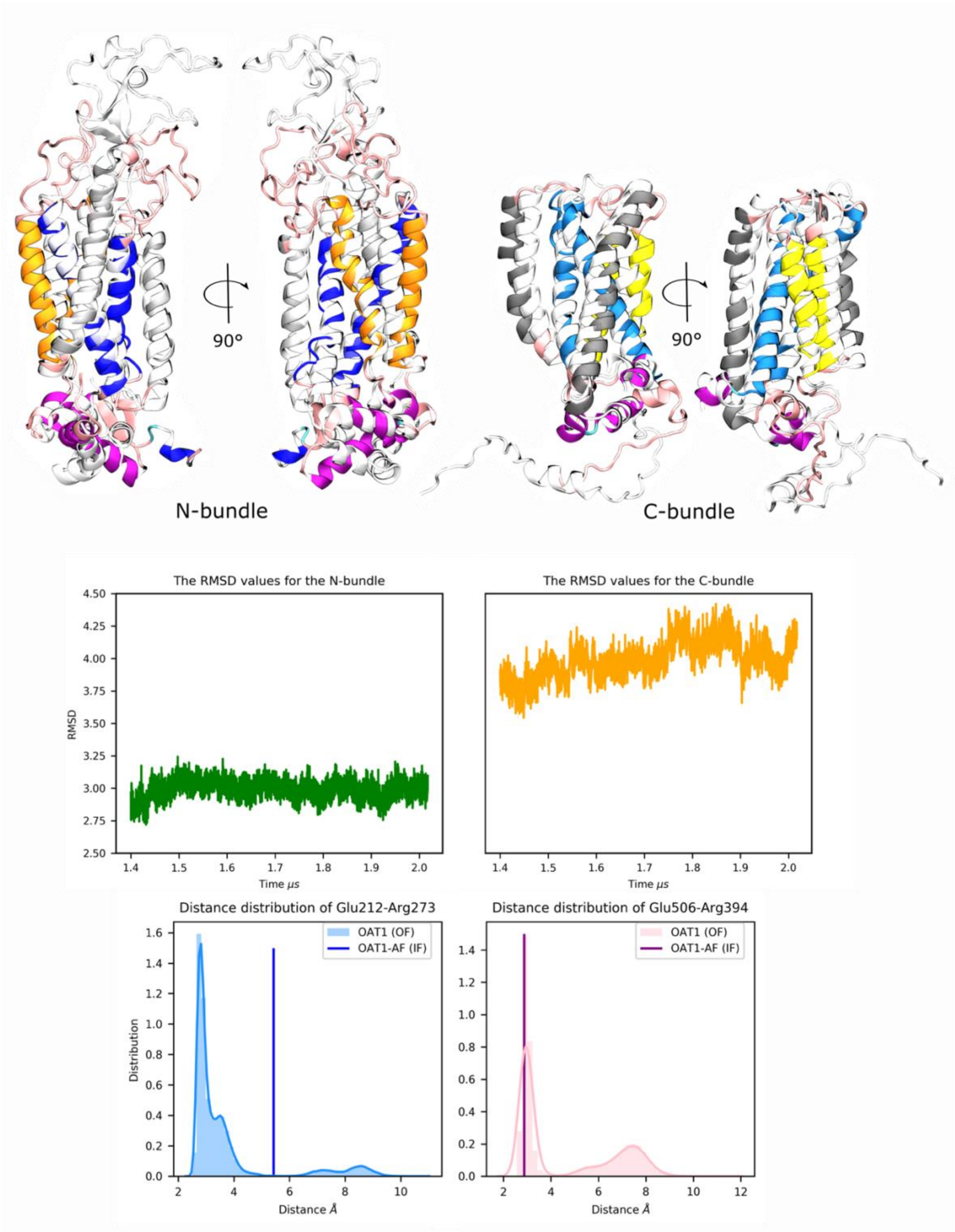

Supplementary Figure S3. The comparison of AF2 (IF) and present study *h*OAT1 (OF) models. The alignment is displayed by a bundle, similarly like RMSD plots below. The crucial distributions for conformation distances are plotted for present *h*OAT1 model with vertical line representing distance for AF2 model.

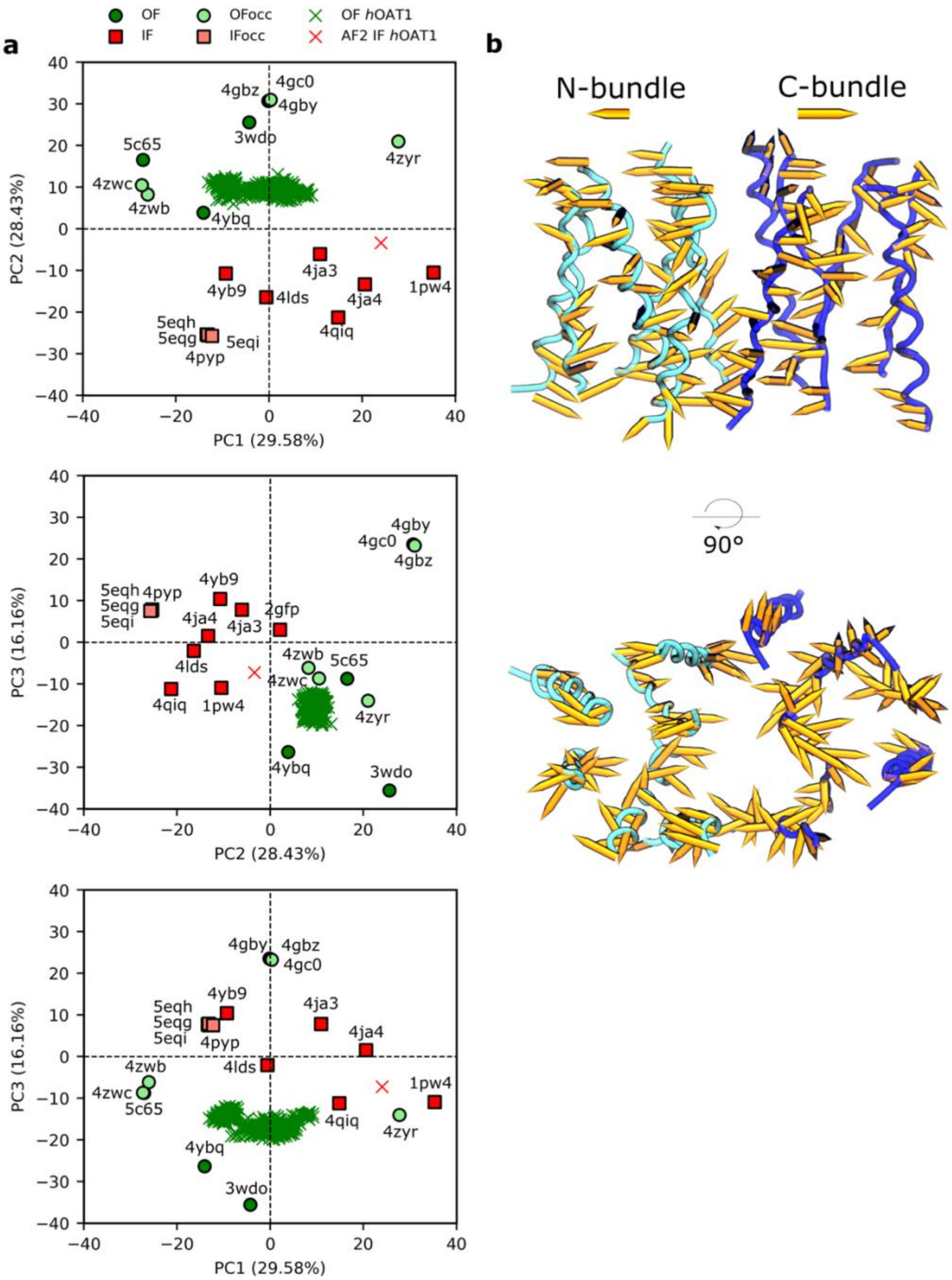

Supplementary Figure S4. (a) *h*OAT1 (both conformations) projected onto MFS conformational space formed by resolved MFS proteins. (b) Porcupine plot presenting PC2 (distinguishes OF and IF conformation) shows higher variability for C-bundle.

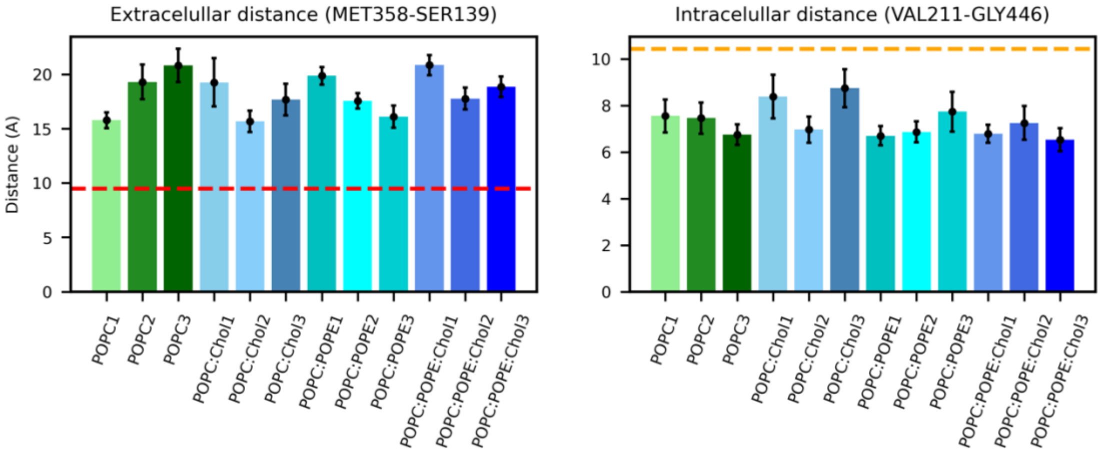

Supplementary Figure S5. Barplot representing distances of extracellular (Met358-Ser139) and intracellular (Val211-Gly446) distances stressed out and compared with previous *h*OAT1 model in IF conformation by Tsigelny *et al.* [24].

(A) POPC:POPE:Chol 2:1:1

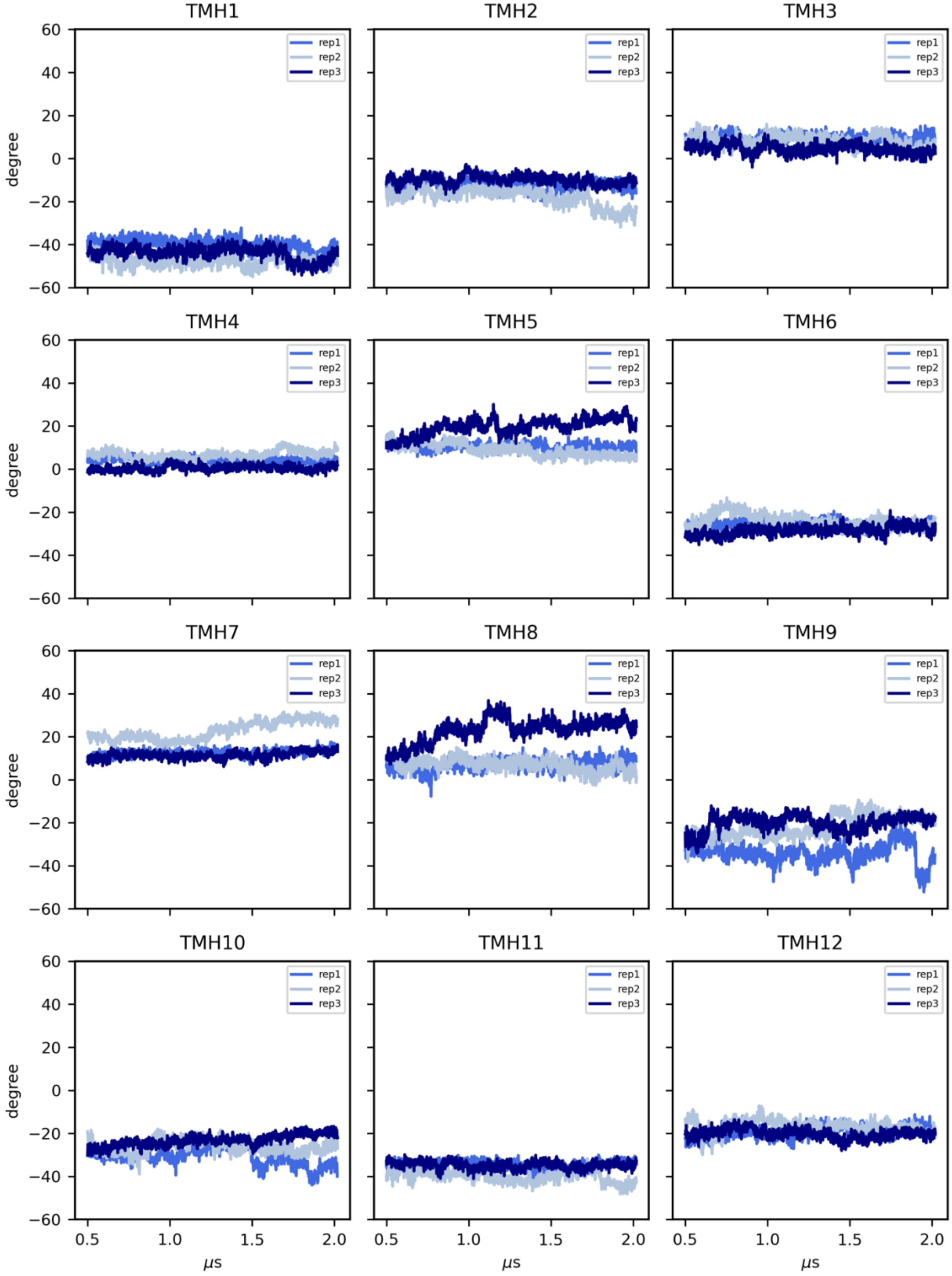

(B) POPC:Chol 3:1

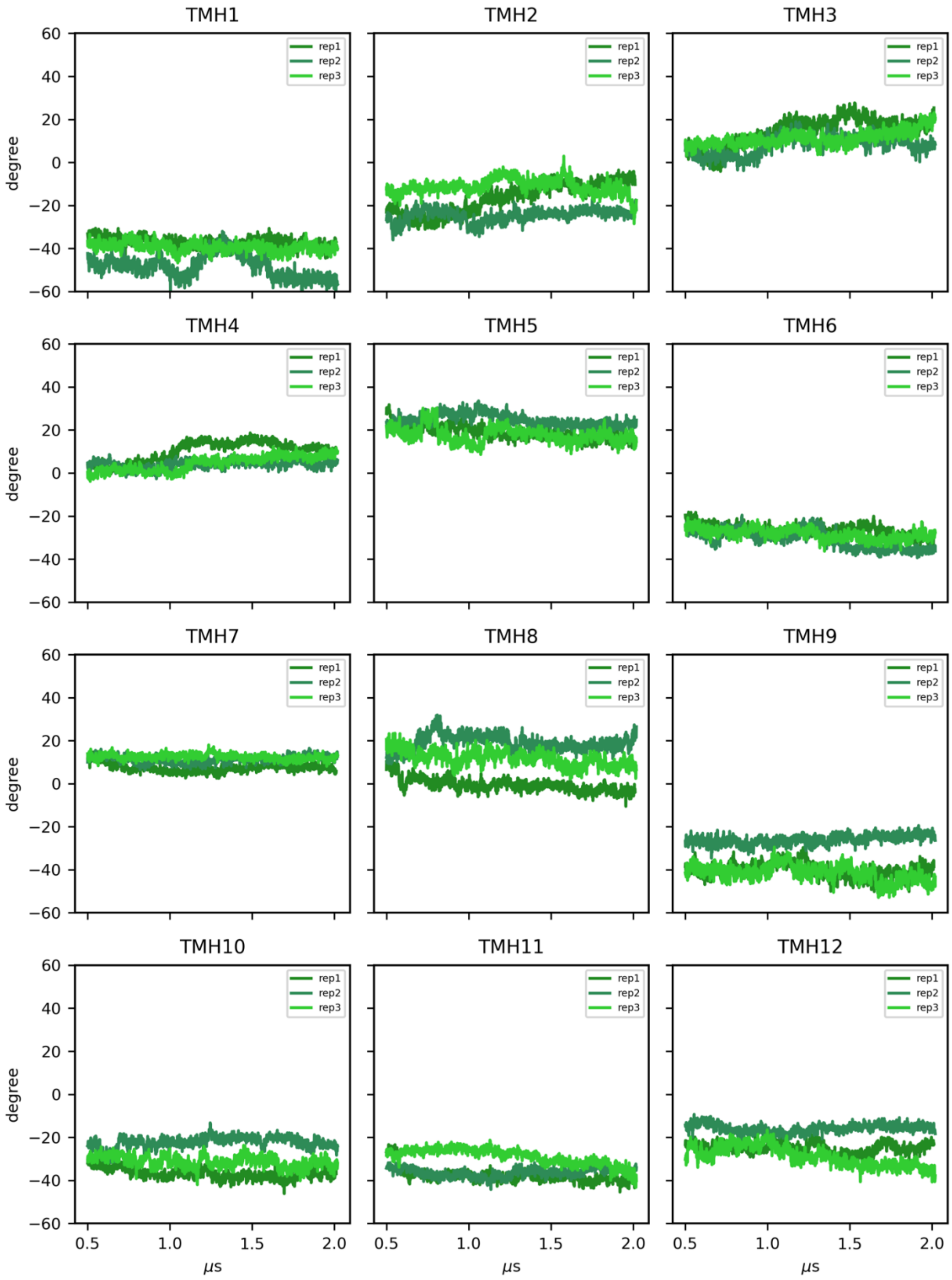

(C) POPC:POPE 3:1

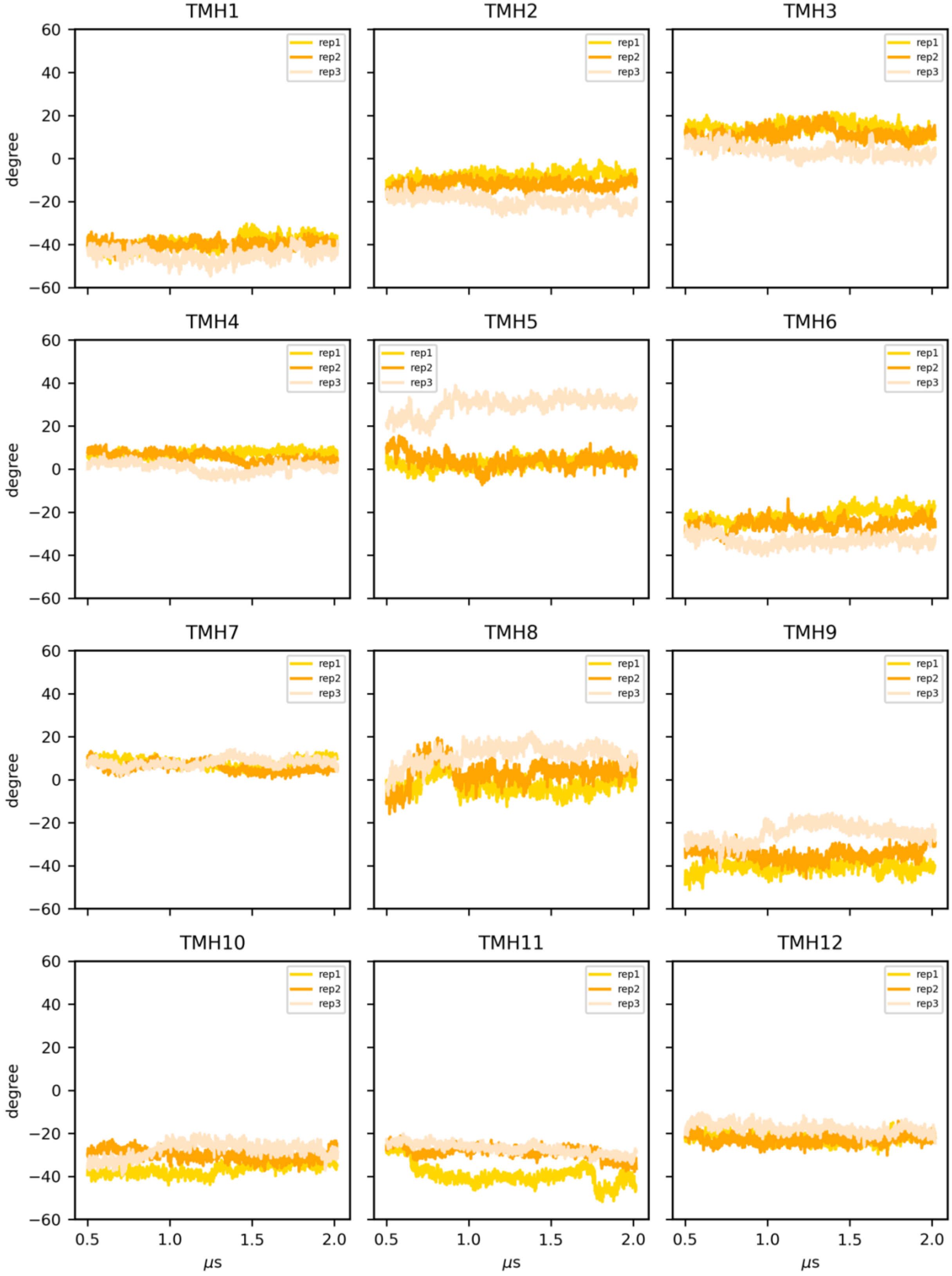

(D) POPC

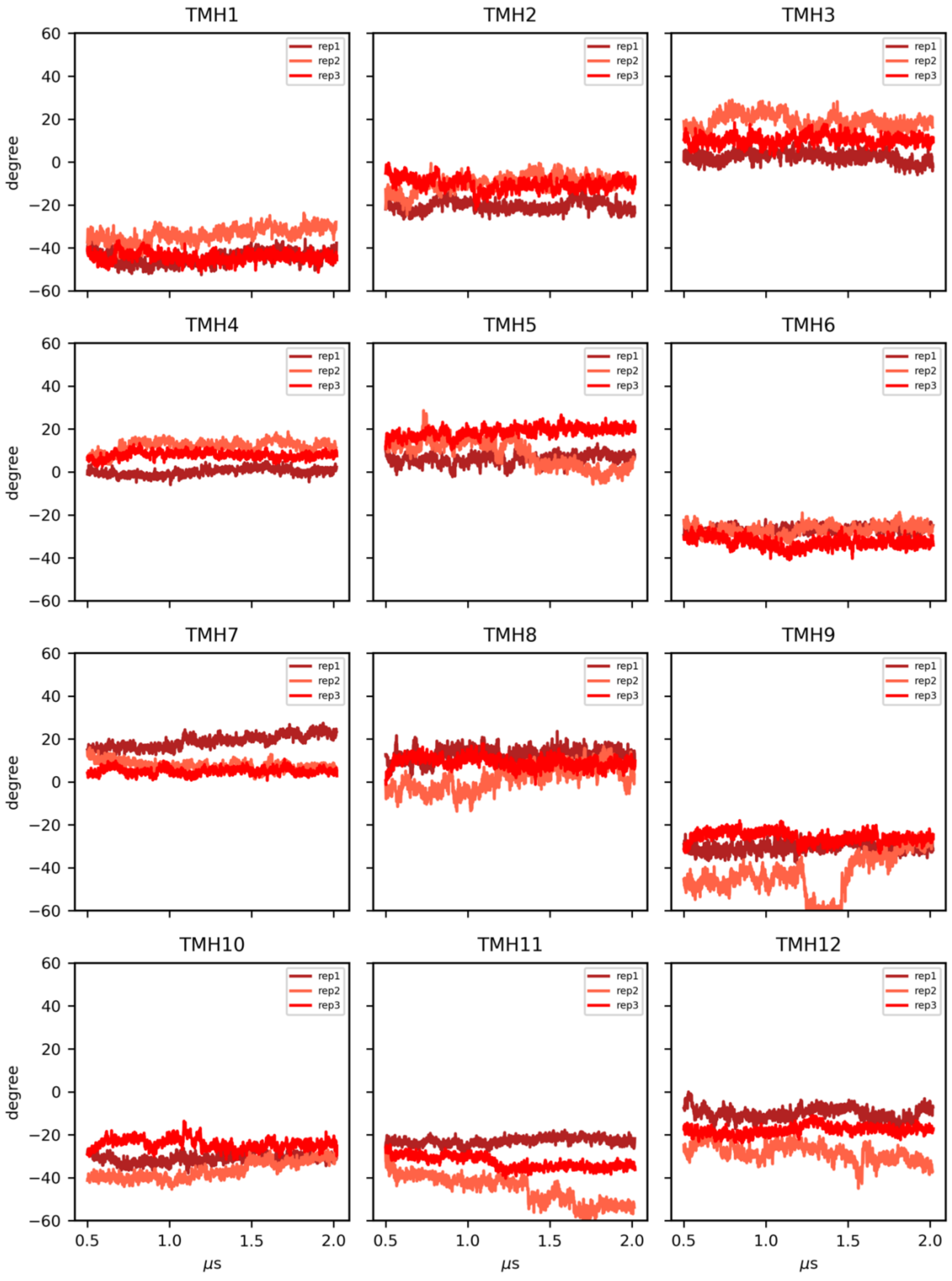

TMH1
TMH2
TMH3
TMH4
TMH5
TMH6
TMH7
TMH8
TMH9
TMH10
TMH11
TMH12
rep1
rep2
rep3
degree
60
40
20
0
−20
−40
−60
0.5
1.0
1.5
2.0
μs

Supplementary Figure S6. The evolution of tilt angles (clock-wise) divided by transmembrane helices of equilibrated trajectory in time for (A) POPC:POPE:Chol (2:1:1), (B) POPC:Chol (3:1), (C) POPC:POPE (3:1), (D) POPC.

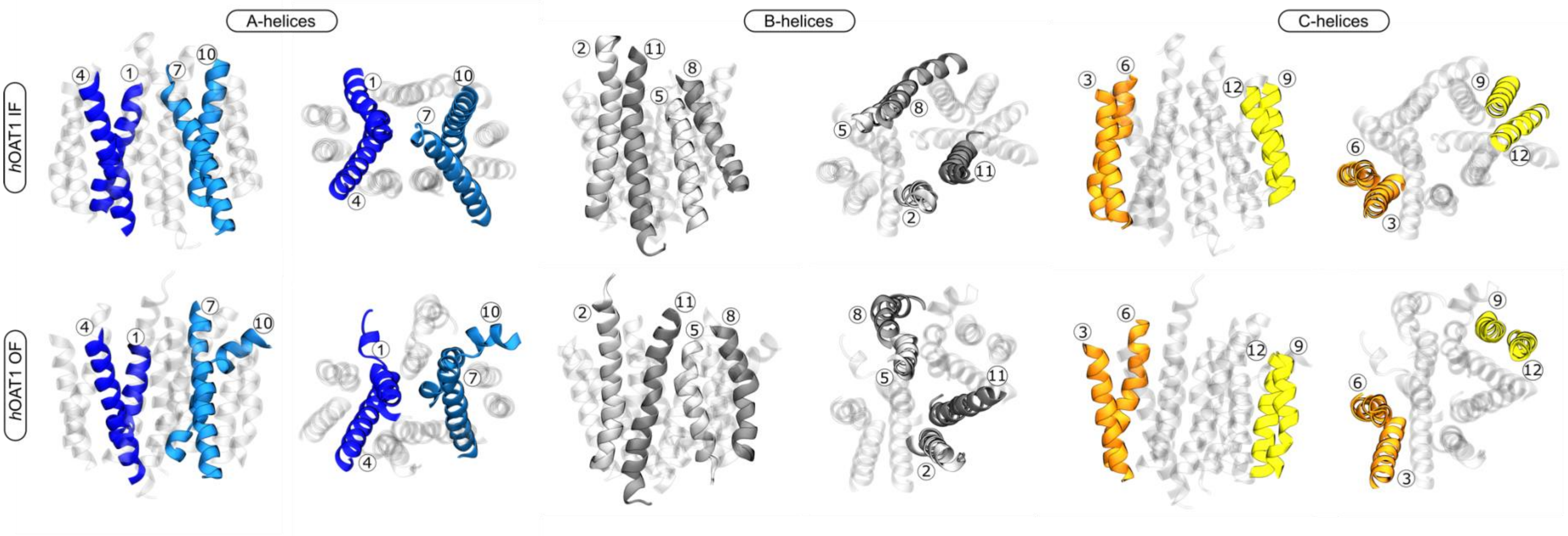

Supplementary Figure S7. Comparison of A-, B-, and C-helices in AF2 (upper) and present study model (bottom) of *h*OAT1.

(A) POPC:POPE:Chol 2:1:1 *h*OAT1 OF

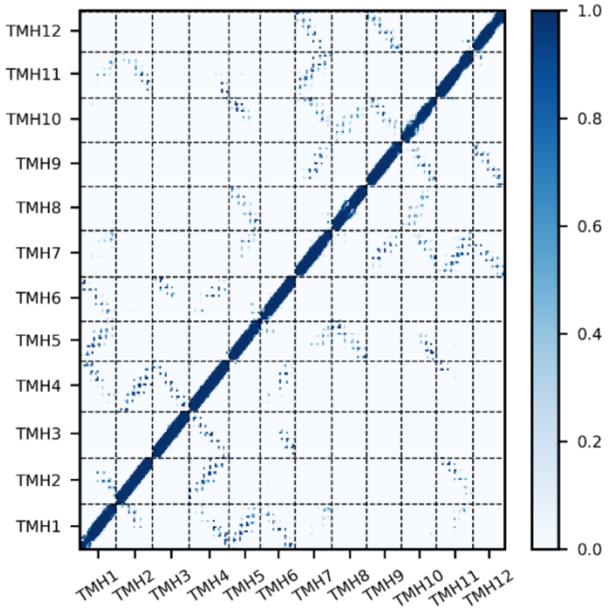

(B) POPC:POPE:Chol 2:1:1 *h*OAT1 IF

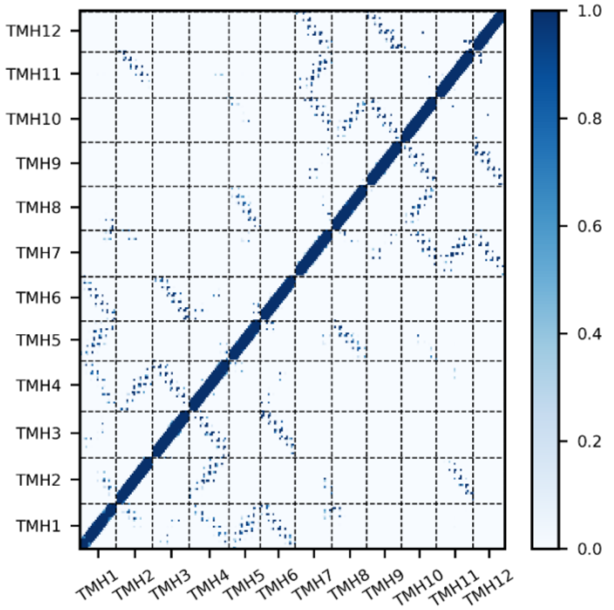

Supplementary Figure S8. The averaged along MD simulations contact maps of *h*OAT1 in (A) OF and (B) IF conformation divided by transmembrane helices.

(A) *h*OAT1 OF in POPC:POPE:Chol

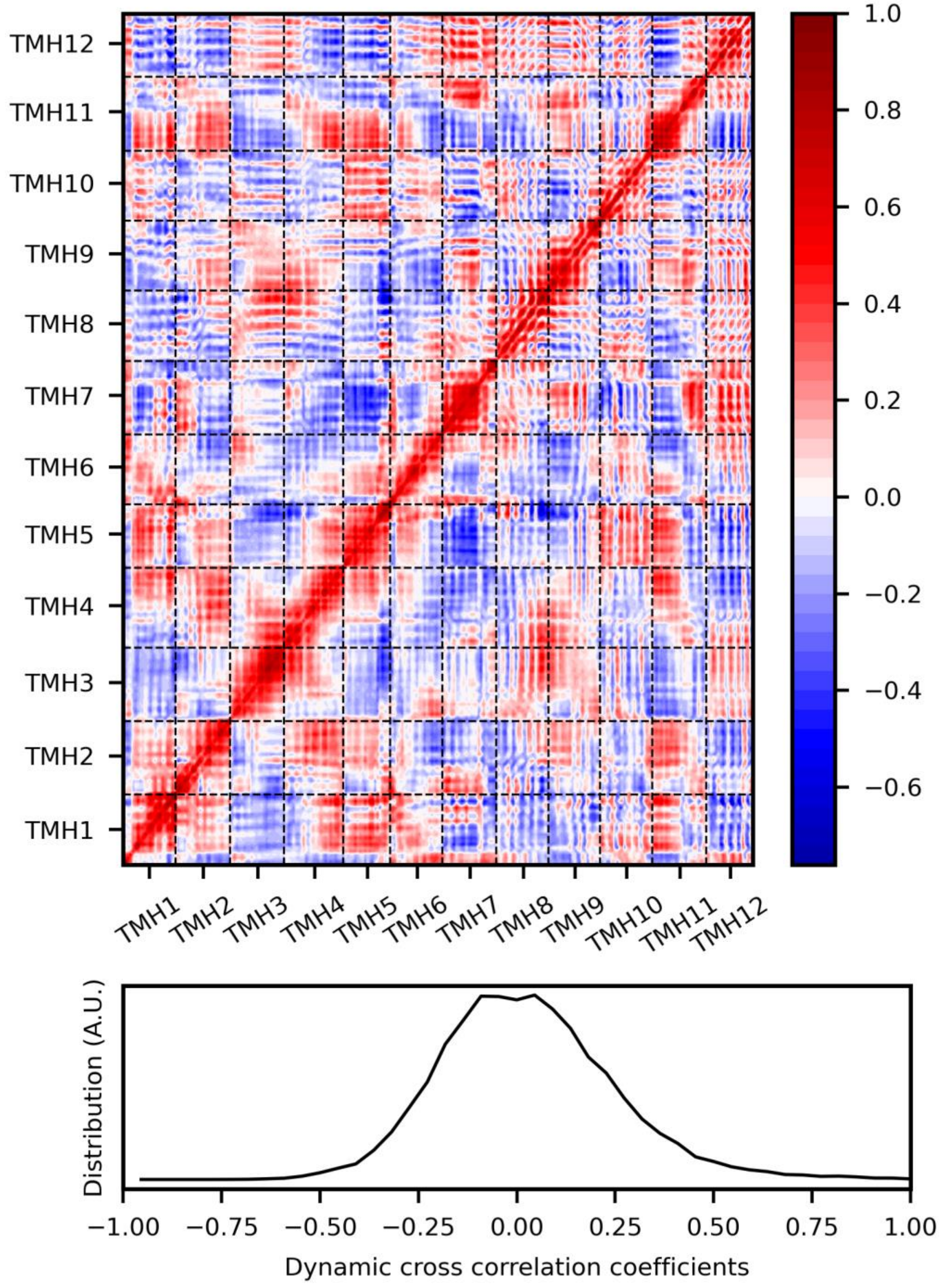

(B) *h*OAT1 IF in POPC:POPE:Chol (2:1:1)

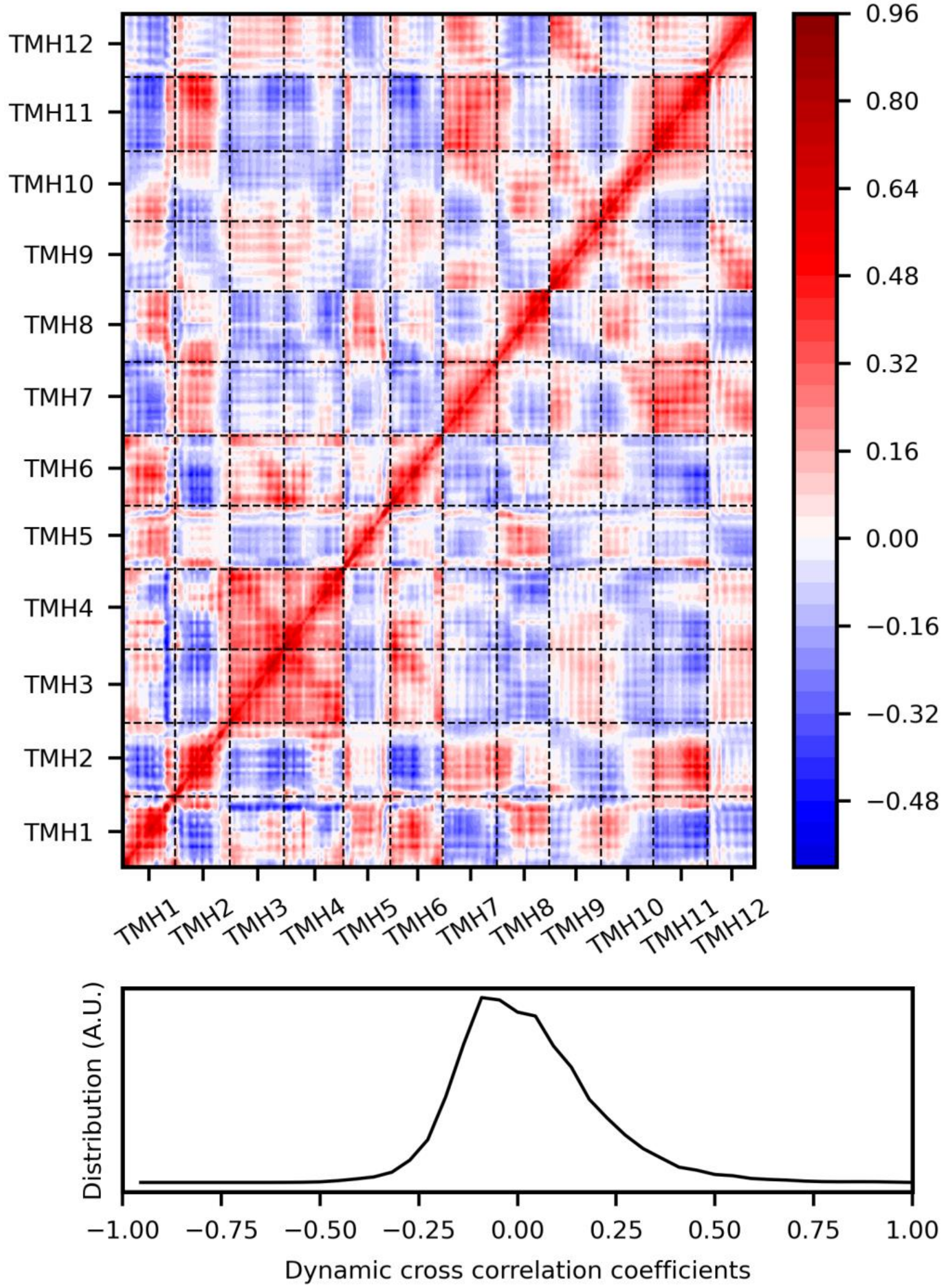

Supplementary Figure S9. Dynamic Cross Correlation Matrix (DCCM) of (A) *h*OAT1 OF and (B) *h*OAT1 IF (in POPC:POPE:Chol 2:1:1) divided by transmembrane helices.

(A) POPC:POPE:Chol (2:1:1)

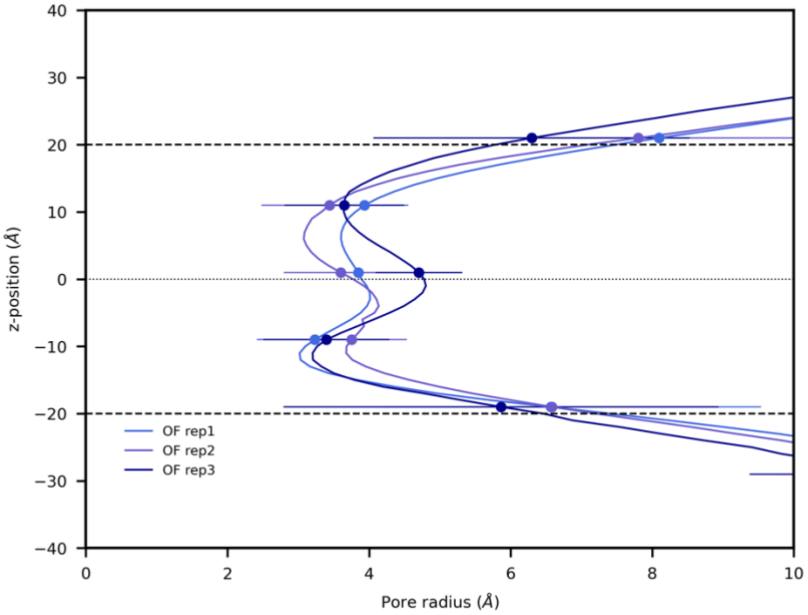

(B) POPC:Chol (3:1)

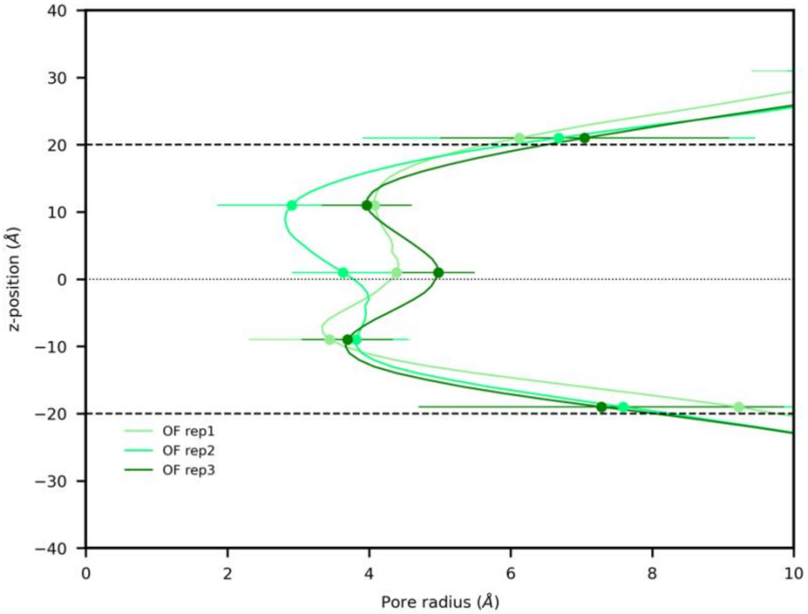

(C) POPC:POPE (3:1)

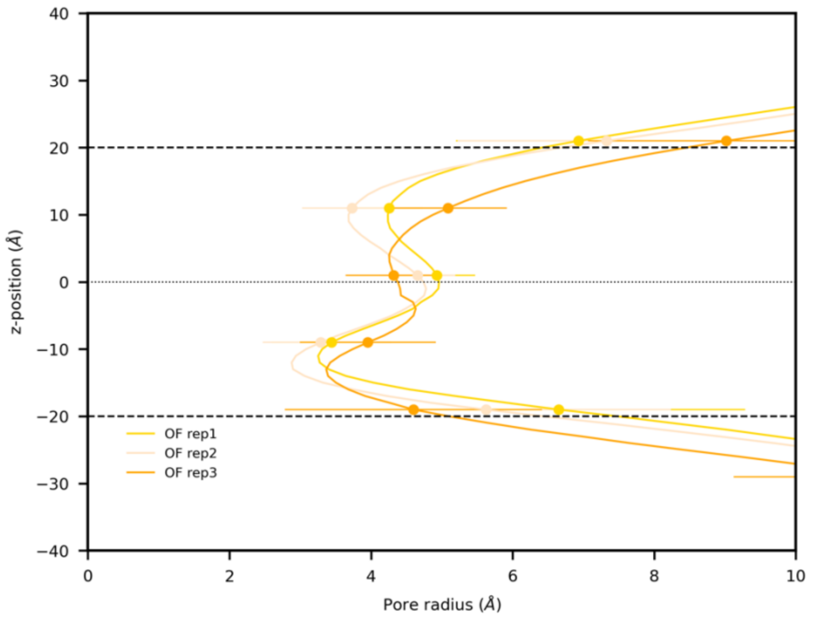

(D) POPC

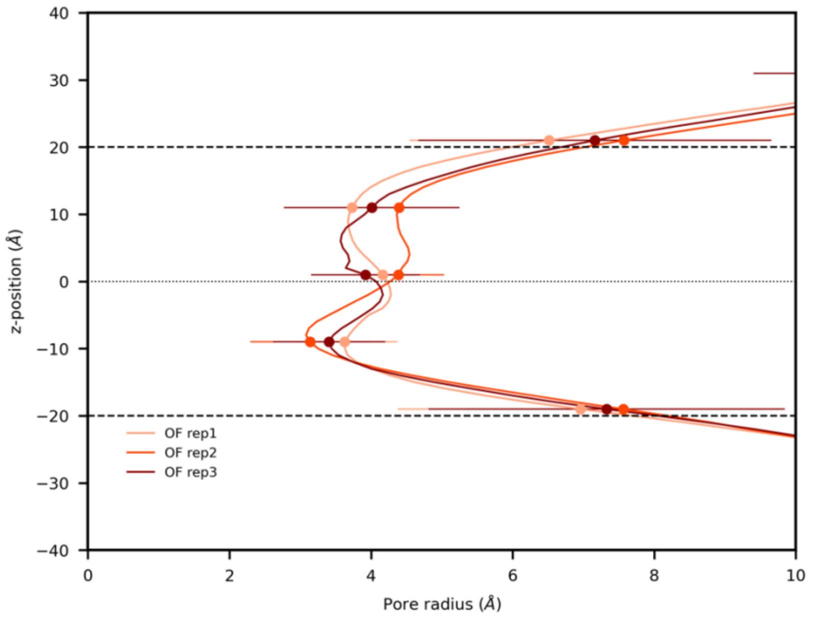

Supplementary Figure S10. Cavity pore radii of *h*OAT1 averaged over 500 snapshots of equilibrated trajectory over z axis for (A) POPC:POPE:Chol (2:1:1); (B) POPC:Chol (3:1); (C) POPC:POPE (3:1); (D) POPC.

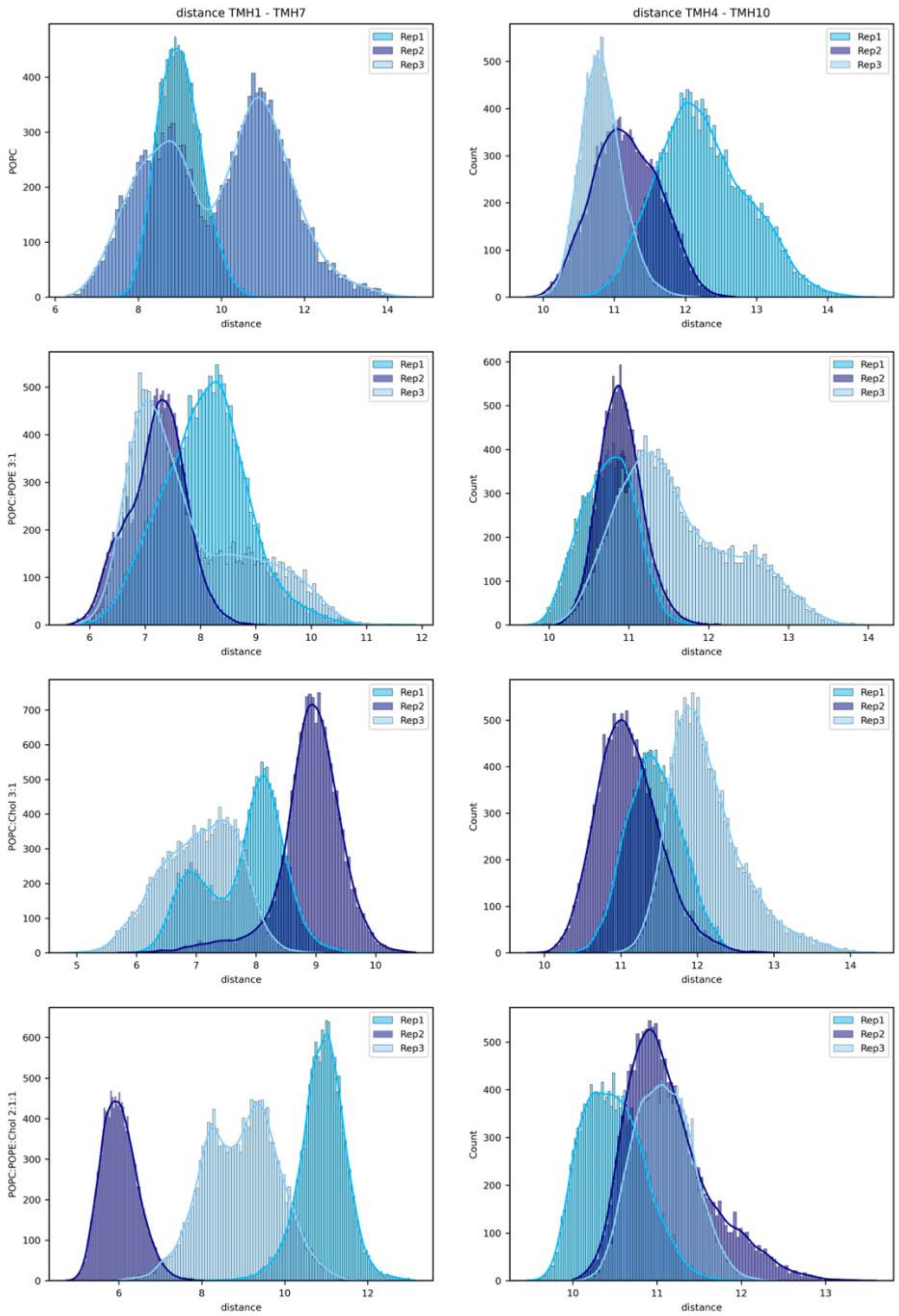

Supplementary Figure S11. Distribution of intra- (TM4-TM10; right column) and extracellular (TM7-TM10; left column) distances. The distances were considered as centers of mass (only backbones) of following residues: 36-40, 354-357 for TMH1-TMH7 and 207-210; 442-445 for TMH4-TMH10.

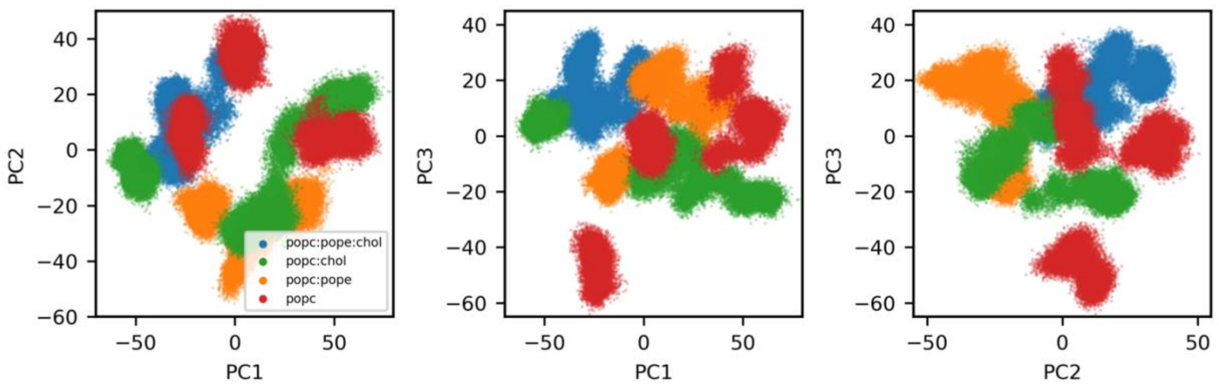

Supplementary Figure S12. Conformational space obtained by PCA. Projected PC1 and PC2 represent 39% and 19% of variability.

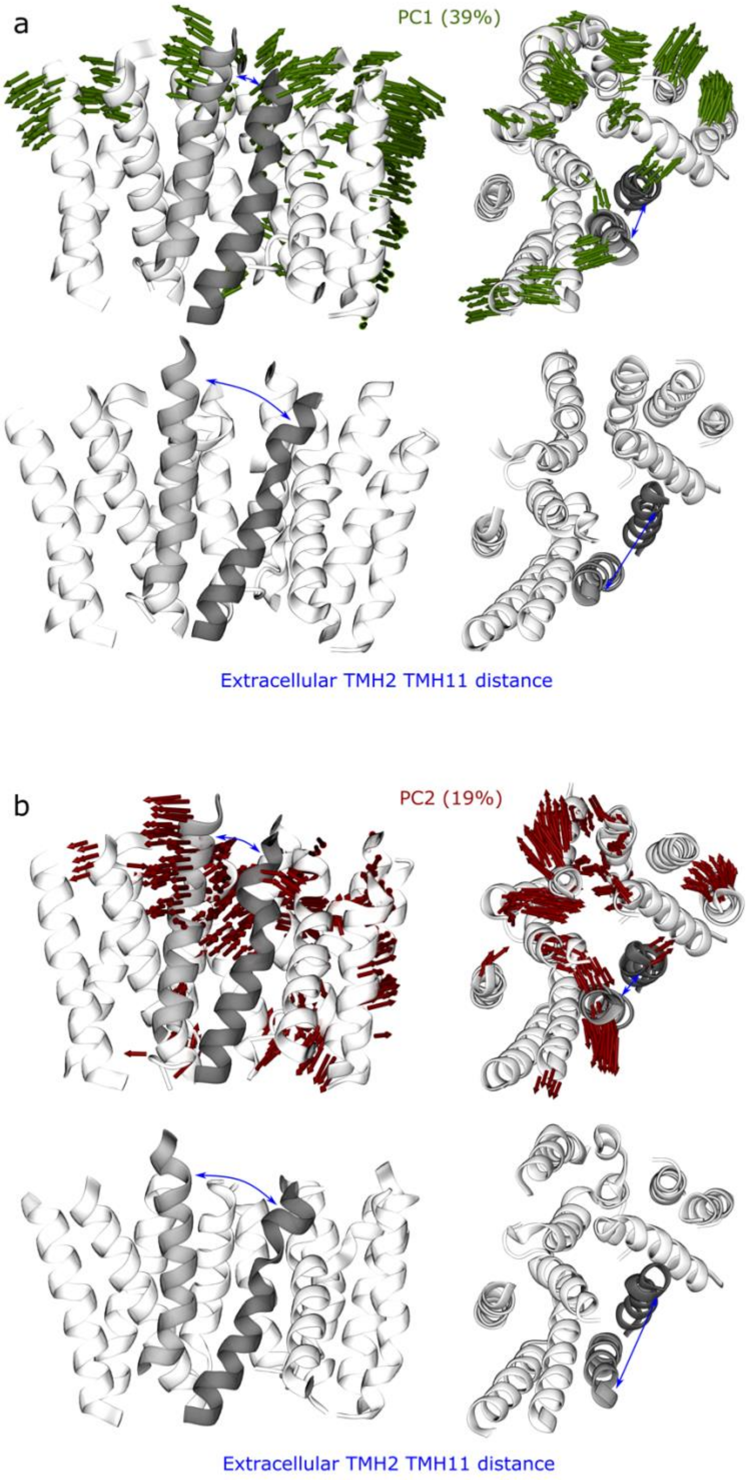

Supplementary Figure S13. Visualization of (a) PC1 and (b) PC2 (from Supplementary Figure S12) representing opening of the extracellular cavity of *h*OAT1. Both PCs display correlated movement of TMH2 (light grey) and TMH11 (dark grey).

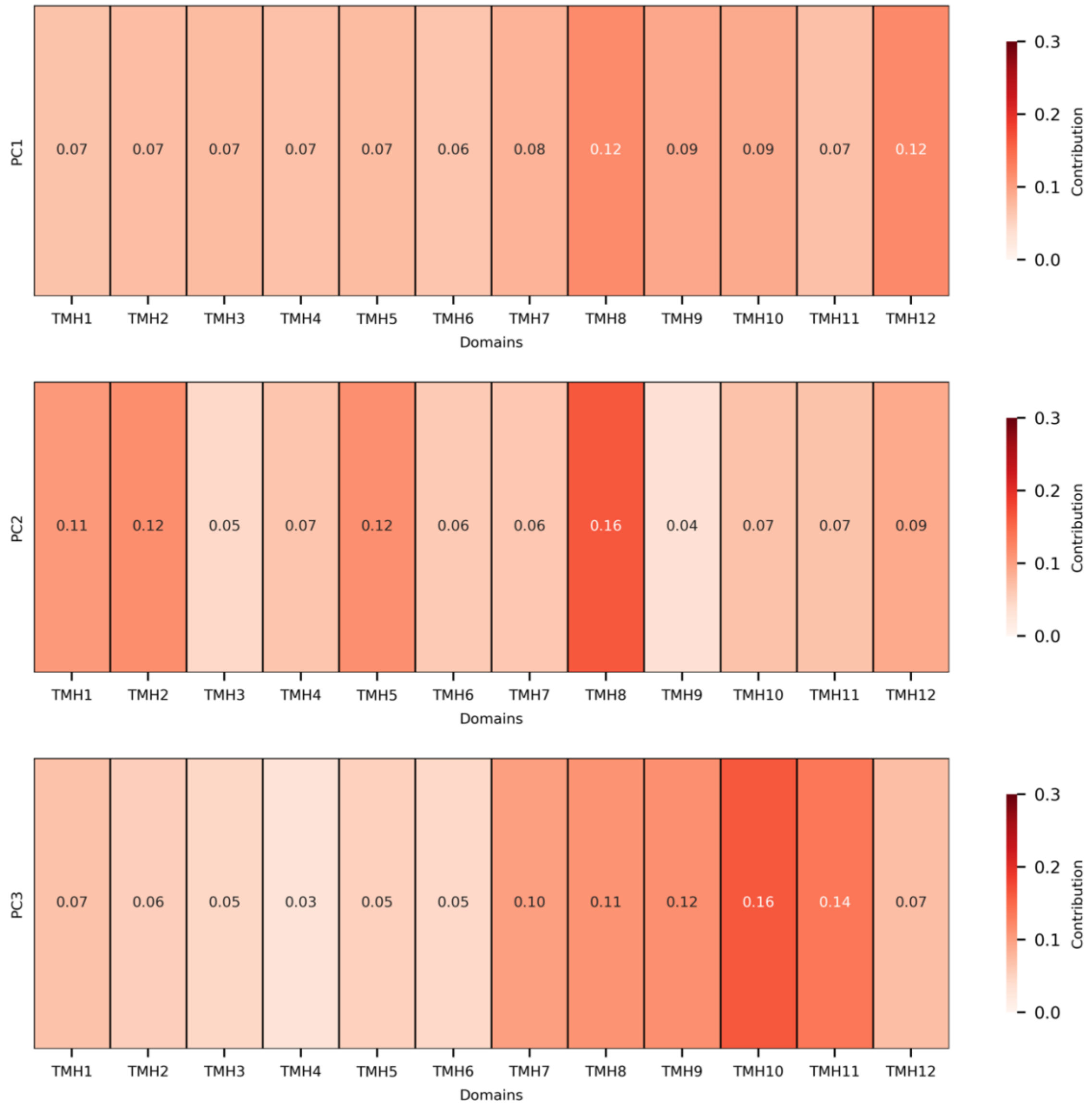

Supplementary Figure S14. The contribution of each TMH into PC1, PC2 and PC3.

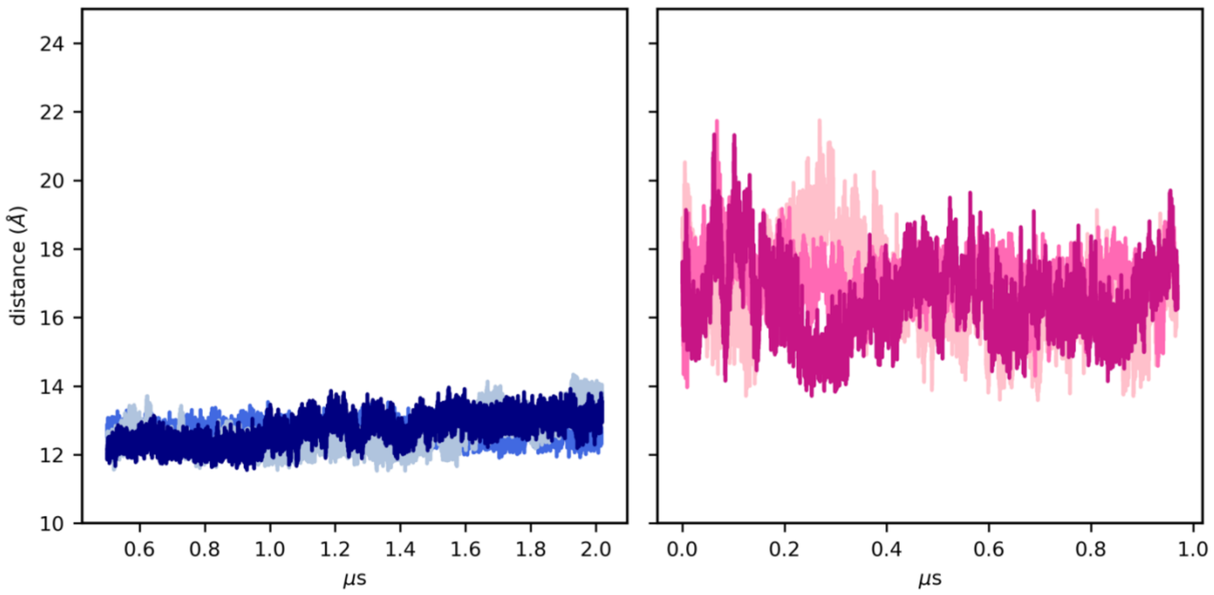

Supplementary Figure S15. Inter-bundle distance of E[X_6]R motifs in N- and C-bundle pictured along MD simulations of OF (left) and IF (right) *h*OAT1.

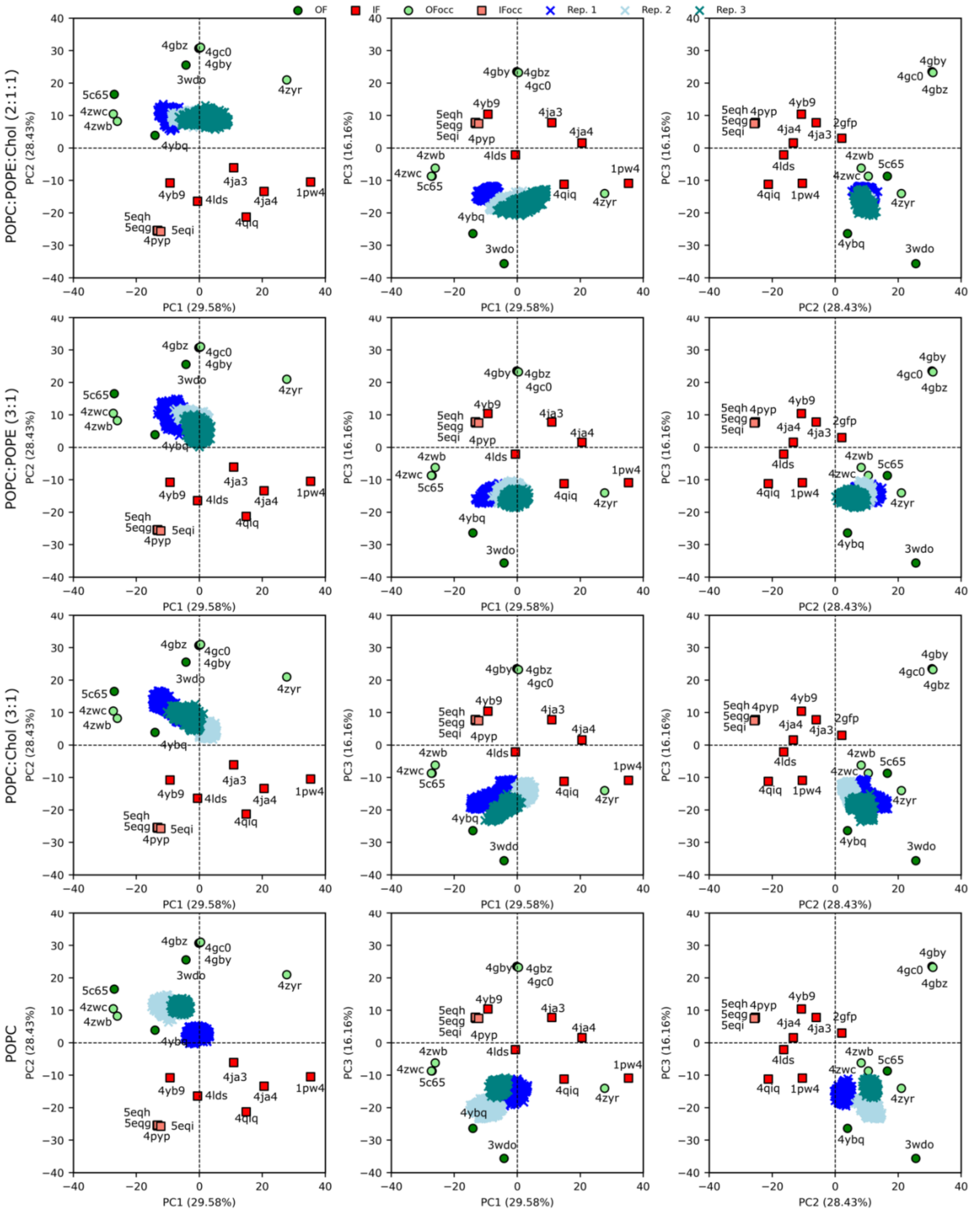

Supplementary Figure S16. The PCA projection of MFS conformational space including *h*OAT1 (OF) models in different lipid bilayers.

(A)

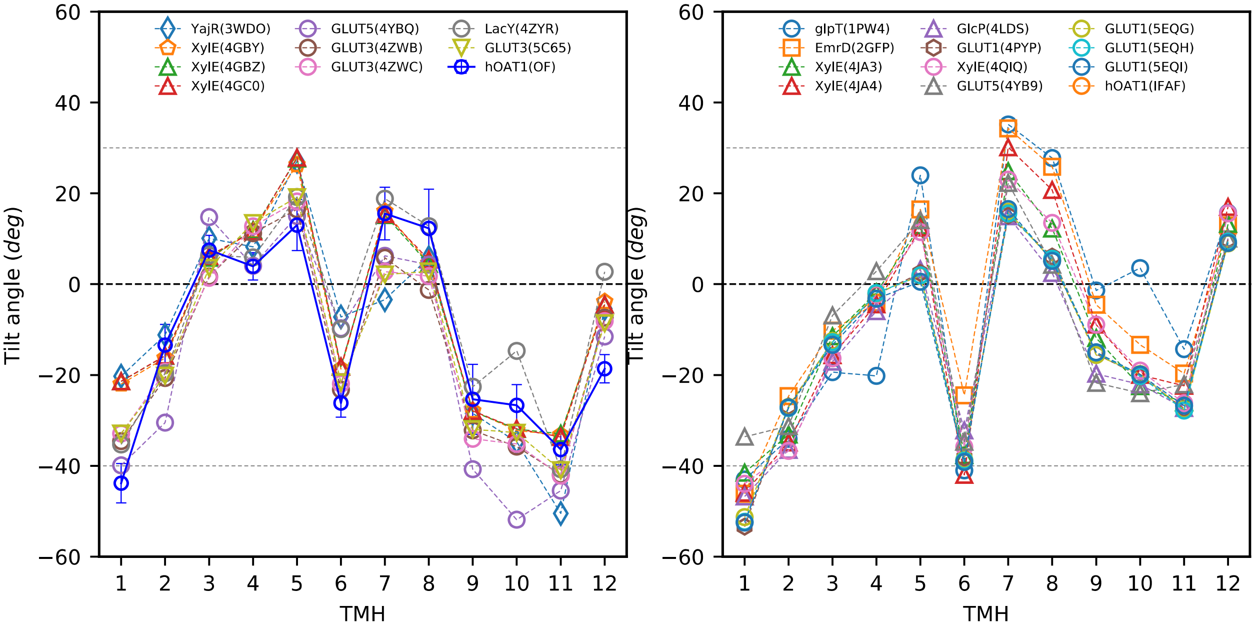

YajR(3WDO)
XylE(4GBY)
XylE(4GBZ)
XylE(4GC0)
GLUT5(4YBQ)
GLUT3(4ZWB)
GLUT3(4ZWC)
LacY(4ZYR)
GLUT3(5C65)
hOAT1(OF)
glpT(1PW4)
EmrD(2GFP)
XylE(4JA3)
XylE(4JA4)
GlcP(4LDS)
GLUT1(4PYP)
XylE(4QIQ)
GLUT5(4YB9)
GLUT1(5EQG)
GLUT1(5EQH)
GLUT1(5EQI)
hOAT1(IFAF)
Tilt angle (deg)
TMH

(B)

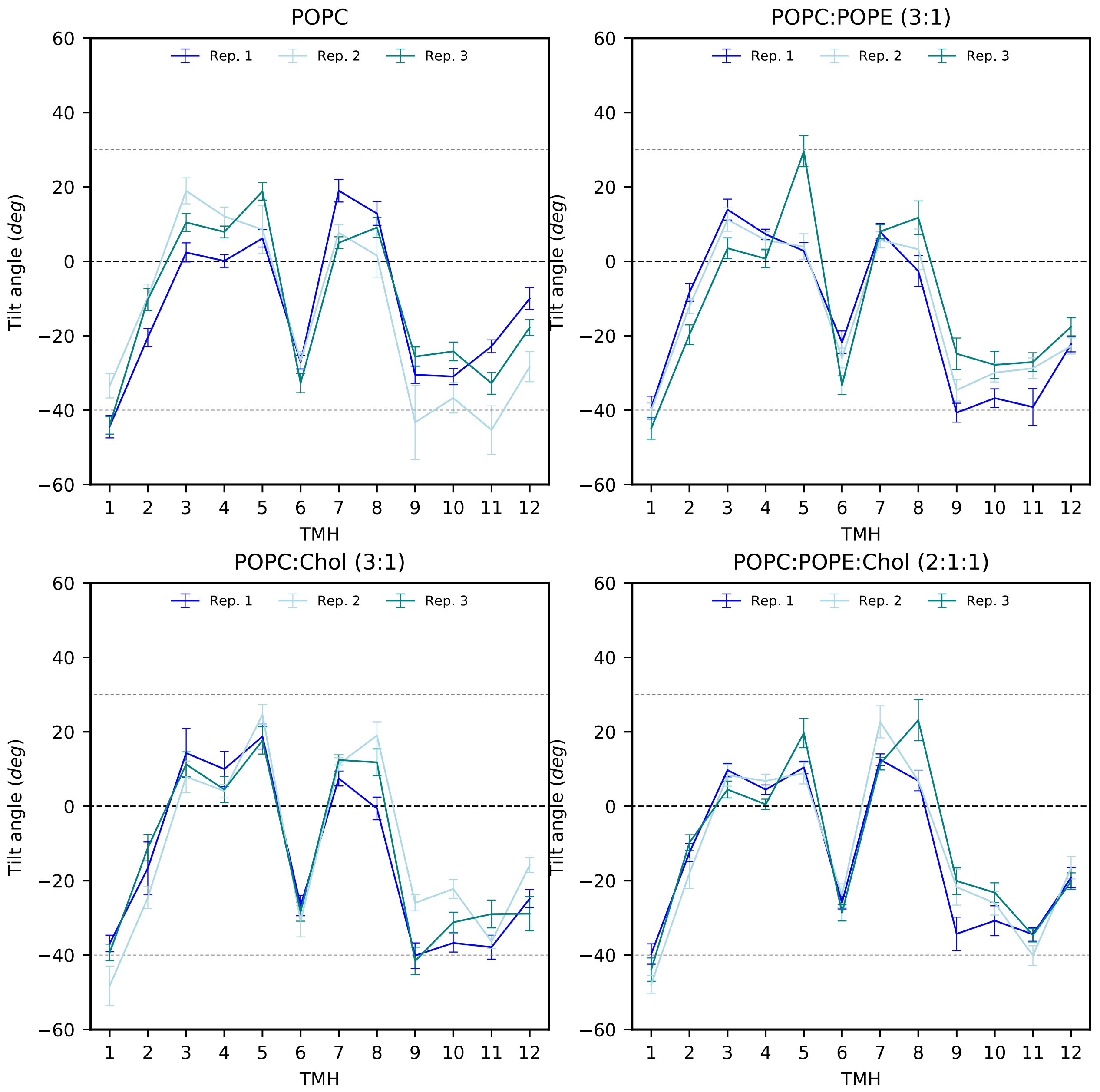

Supplementary Figure S17. The tilt angle profile of (A) resolved structures of MFS transporters (including occluded states); (B) *h*OAT1 OF (averaged over MD simulations) in different lipid bilayers.

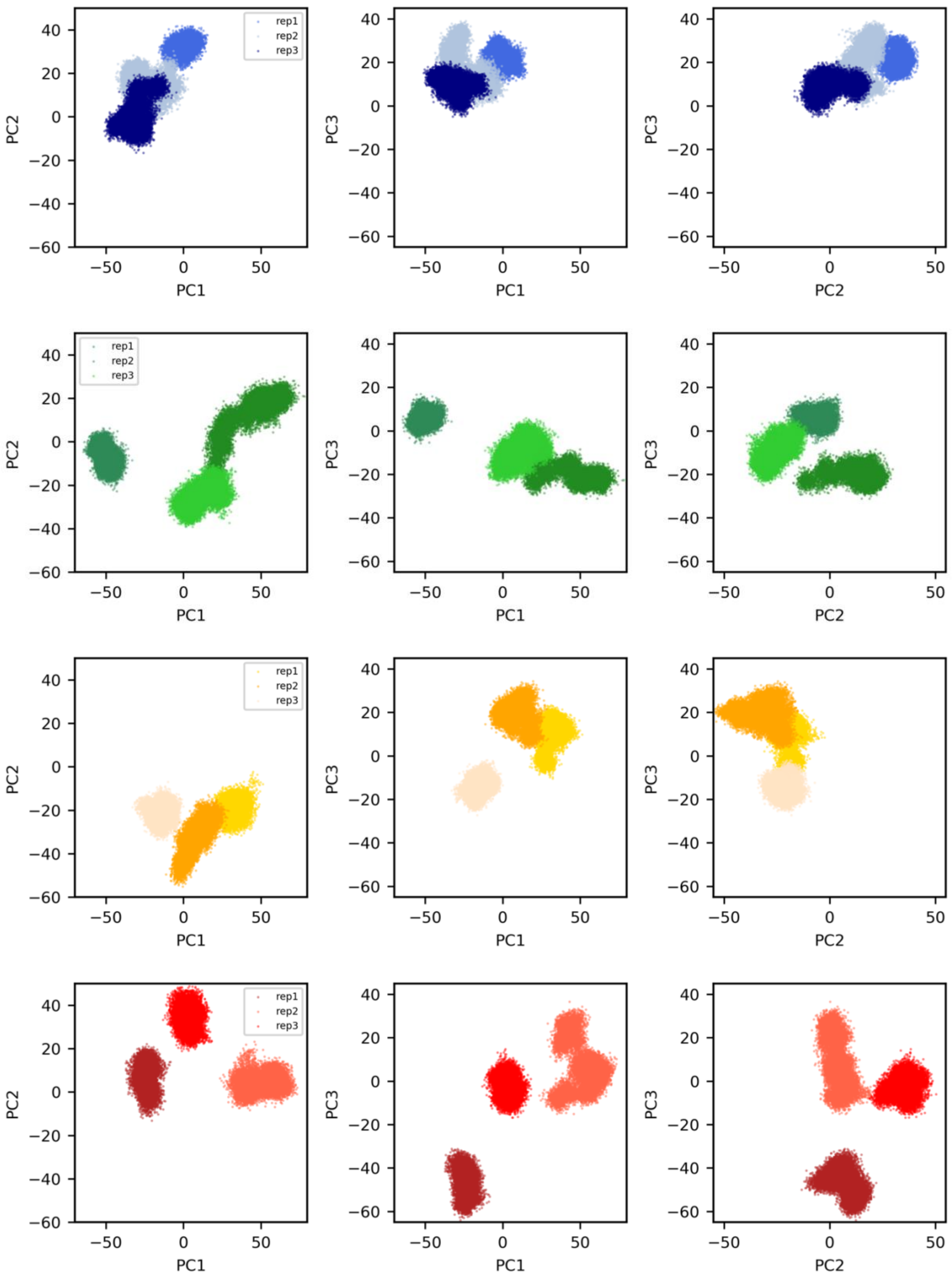
rep1
rep2
rep3
PC1
PC2
PC3

Supplementary Figure S18. PCA projected on all membrane conformational space of *h*OAT1 embedded in POPC:POPE:Chol (2:1:1) (blues), POPC:Chol (3:1) (greens), POPC:POPE (3:1) (yellows) and POPC (reds)

(A)

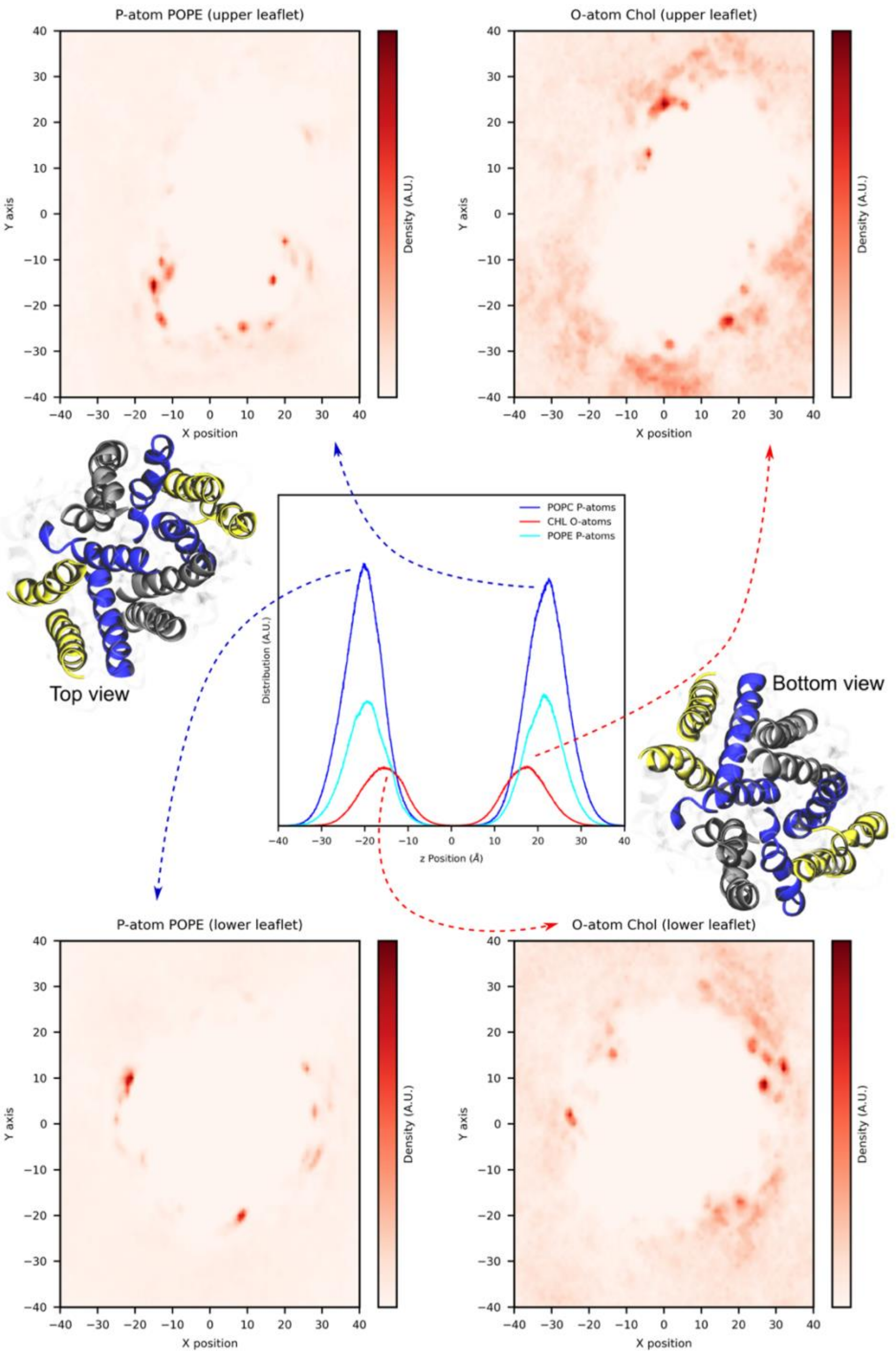
P-atom POPE (upper leaflet)
O-atom Chol (upper leaflet)
P-atom POPE (lower leaflet)
O-atom Chol (lower leaflet)
Y axis
X position
Density (A.U.)
POPC P-atoms
CHL O-atoms
POPE P-atoms
Distribution (A.U.)
z Position (Å)
Top view
Bottom view

(B)

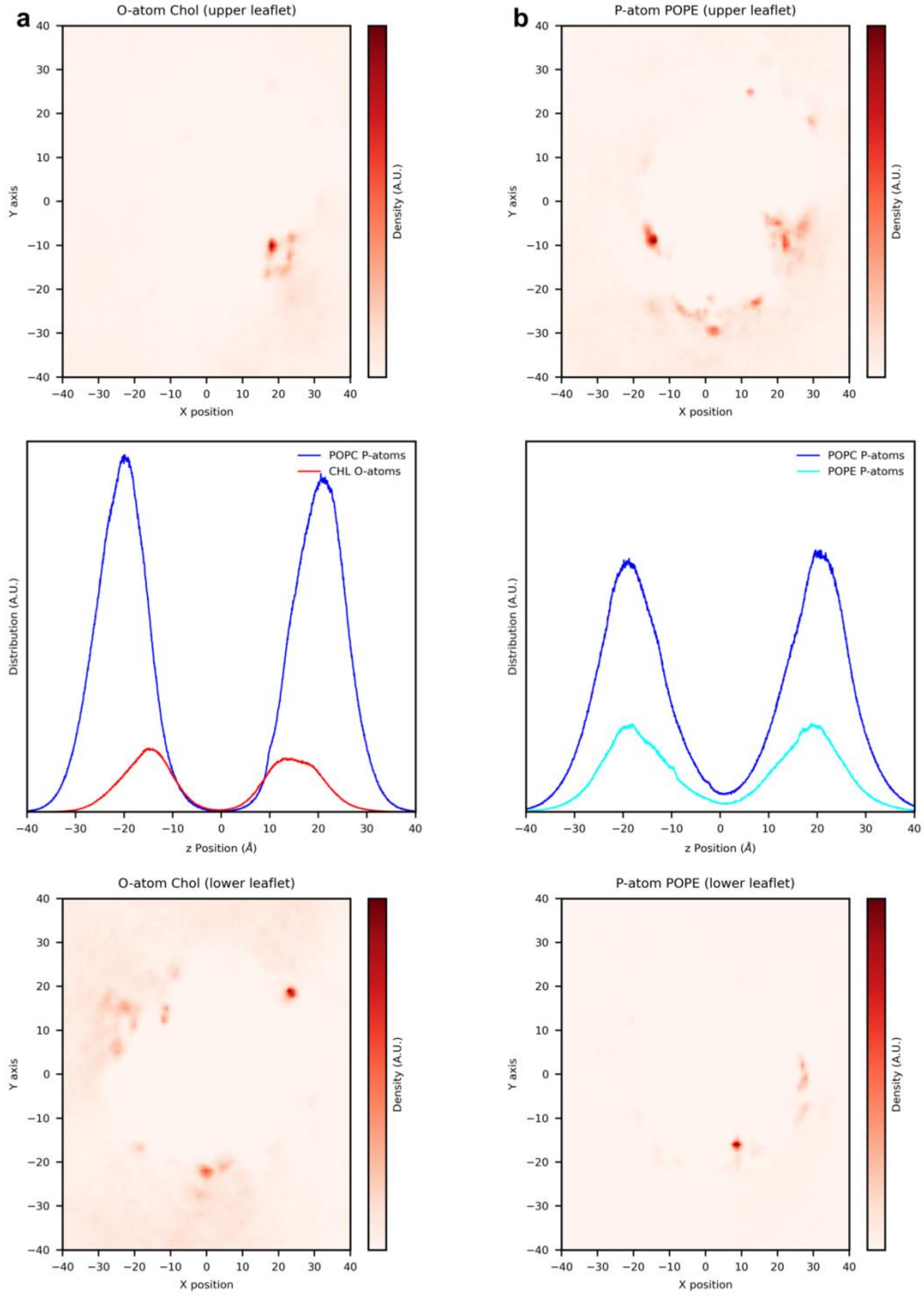

Supplementary Figure S19. Lipid density maps of *h*OAT1 inserted into (A) POPC:POPE:Chol (2:1:1). Upper and bottom leaflet of POPE (on the left) and cholesterol (on the right) are represented as density of P and O atom, respectively. (B) Lipid density maps of *h*OAT1 inserted into POPC:Chol (3:1) and POPC:POPE (3:1), shown with subpanel a and b, respectively.

3. References

1. Nomura, N. *et al.* Structure and mechanism of the mammalian fructose transporter GLUT5. *Nature* **526**, 397–401 (2015).
2. Kumar, H., Finer-Moore, J. S., Kaback, H. R. & Stroud, R. M. Structure of LacY with an α-substituted galactoside: Connecting the binding site to the protonation site. *Proc. Natl. Acad. Sci.* **112**, 9004–9009 (2015).
3. Sun, L. *et al.* Crystal structure of a bacterial homologue of glucose transporters GLUT1–4. *Nature* **490**, 361–366 (2012).
4. Quistgaard, E. M., Löw, C., Moberg, P., Trésaugues, L. & Nordlund, P. Structural basis for substrate transport in the GLUT-homology family of monosaccharide transporters. *Nat. Struct. Mol. Biol.* **20**, 766–768 (2013).
5. Wisedchaisri, G., Park, M.-S., Iadanza, M. G., Zheng, H. & Gonen, T. Proton-coupled sugar transport in the prototypical major facilitator superfamily protein XylE. *Nat. Commun.* **5**, 4521 (2014).
6. Deng, D. *et al.* Crystal structure of the human glucose transporter GLUT1. *Nature* **510**, 121–125 (2014).
7. Kapoor, K. *et al.* Mechanism of inhibition of human glucose transporter GLUT1 is conserved between cytochalasin B and phenylalanine amides. *Proc. Natl. Acad. Sci.* **113**, 4711–4716 (2016).
8. Deng, D. *et al.* Molecular basis of ligand recognition and transport by glucose transporters. *Nature* **526**, 391–396 (2015).

9. Iancu, C. V., Zamoon, J., Woo, S. B., Aleshin, A. & Choe, J. -y. Crystal structure of a glucose/H+ symporter and its mechanism of action. *Proc. Natl. Acad. Sci.* **110**, 17862–17867 (2013).

10. Huang, Y. Structure and Mechanism of the Glycerol-3-Phosphate Transporter from Escherichia coli. *Science* **301**, 616–620 (2003).

11. Jiang, D. *et al.* Structure of the YajR transporter suggests a transport mechanism based on the conserved motif A. *Proc. Natl. Acad. Sci.* **110**, 14664–14669 (2013).

12. Yin, Y. Structure of the Multidrug Transporter EmrD from Escherichia coli. *Science* **312**, 741–744 (2006).

13. Jumper, J. *et al.* Highly accurate protein structure prediction with AlphaFold. *Nature* (2021) doi:10.1038/s41586-021-03819-2.

14. Hong, M., Zhou, F. & You, G. Critical Amino Acid Residues in Transmembrane Domain 1 of the Human Organic Anion Transporter hOAT1. *J. Biol. Chem.* **279**, 31478–31482 (2004).

15. Tanaka, K., Xu, W., Zhou, F. & You, G. Role of glycosylation in the organic anion transporter OAT1. *J. Biol. Chem.* **279**, 14961–14966 (2004).

16. Perry, J. L., Dembla-Rajpal, N., Hall, L. A. & Pritchard, J. B. A Three-dimensional Model of Human Organic Anion Transporter 1: AROMATIC AMINO ACIDS REQUIRED FOR SUBSTRATE TRANSPORT. *J. Biol. Chem.* **281**, 38071–38079 (2006).

17. Hong, M., Li, S., Zhou, F., Thomas, P. E. & You, G. Putative Transmembrane Domain 12 of the Human Organic Anion Transporter hOAT1 Determines Transporter Stability and Maturation Efficiency. *J. Pharmacol. Exp. Ther.* **332**, 650–658 (2010).

18. Li, S., Zhang, Q. & You, G. Three ubiquitination sites of organic anion transporter-1 synergistically mediate protein kinase C-dependent endocytosis of the transporter. *Mol. Pharmacol.* **84**, 139–146 (2013).

19. Rizwan, A. N., Krick, W. & Burckhardt, G. The chloride dependence of the human organic anion transporter 1 (hOAT1) is blunted by mutation of a single amino acid. *J. Biol. Chem.* **282**, 13402–13409 (2007).

20. Hong, M., Zhou, F., Lee, K. & You, G. The putative transmembrane segment 7 of human organic anion transporter hOAT1 dictates transporter substrate binding and stability. *J. Pharmacol. Exp. Ther.* **320**, 1209–1215 (2007).

21. Xu, W., Tanaka, K., Sun, A. & You, G. Functional Role of the C Terminus of Human Organic Anion Transporter hOAT1. *J. Biol. Chem.* **281**, 31178–31183 (2006).

22. Astorga, B., Wunz, T. M., Morales, M., Wright, S. H. & Pelis, R. M. Differences in the substrate binding regions of renal organic anion transporters 1 (OAT1) and 3 (OAT3). *Am. J. Physiol. Renal Physiol.* **301**, F378-386 (2011).

23. Tanaka, K., Zhou, F., Kuze, K. & You, G. Cysteine residues in the organic anion transporter mOAT1. *Biochem. J.* **380**, 283–287 (2004).

24. Tsigelny, I. F. *et al.* Conformational Changes of the Multispecific Transporter Organic Anion Transporter 1 (OAT1/SLC22A6) Suggests a Molecular Mechanism for Initial Stages of Drug and Metabolite Transport. *Cell Biochem. Biophys.* **61**, 251–259 (2011).
